# Supplementary material for: Single-cell 3D genome imaging shows super-enhancer hubs are rare, nonspecific, and neither necessary nor sufficient for transcriptional bursting
Source: Cell Genom. 2026 Aug 17;6(10):101331. doi: 10.1016/j.xgen.2026.101331 (PMC13689515; doi:10.1016/j.xgen.2026.101331)
Supplement: Supplementary file 1 [file mmc1.pdf]

Cell Genomics, Volume *6*

Supplemental information

Single-cell 3D genome imaging shows super-enhancer hubs are rare, nonspecific, and neither necessary nor sufficient for transcriptional bursting

Derek J. Le, Antonina Hafner, Achuthan Raja Venkatesh, Tee Udomlumleart, Sadhana Gaddam, Ana Novacic, Kevin C. Wang, and Alistair N. Boettiger

Figure S1, related to Figure 1

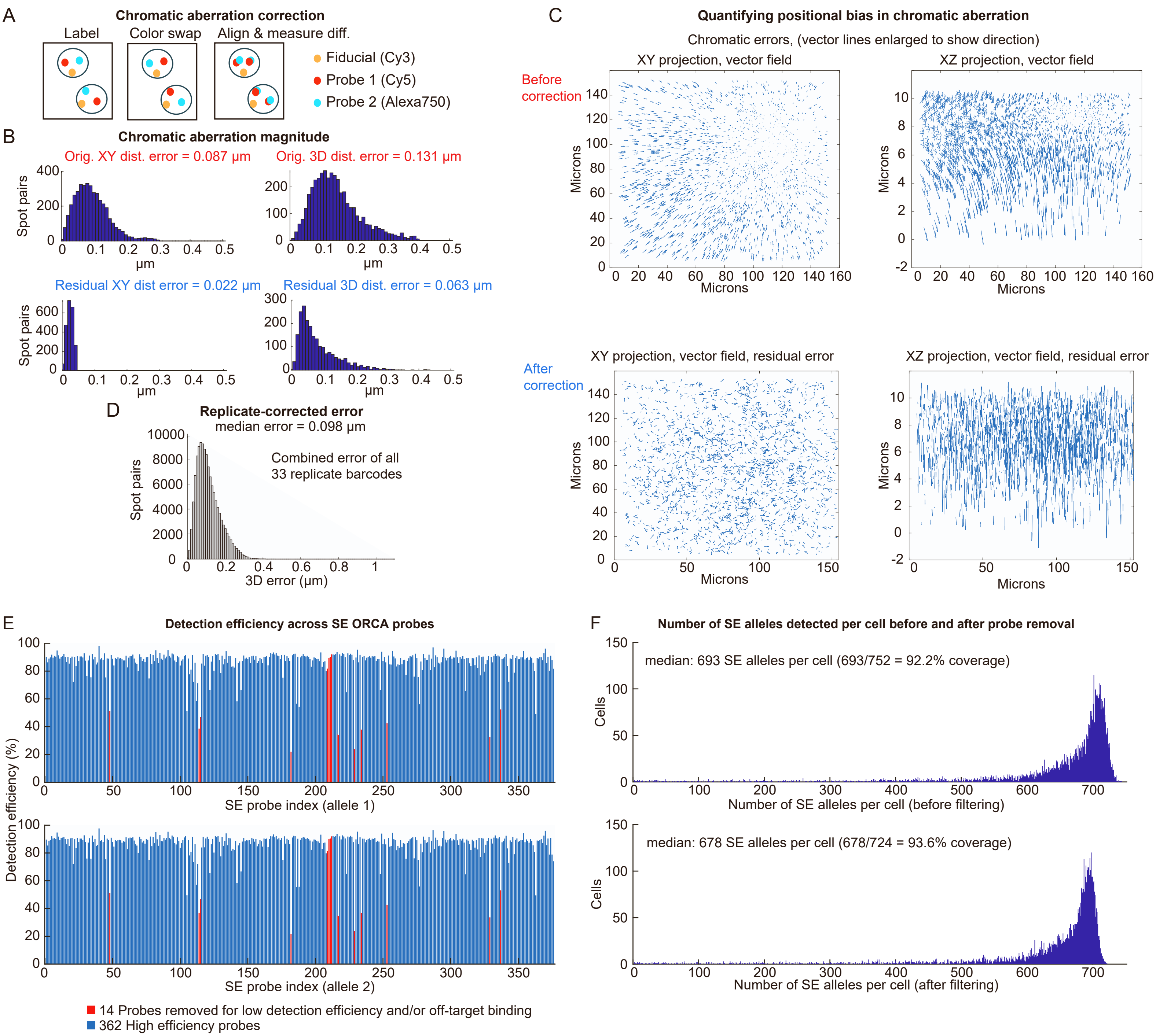

Figure S1 | Quantification of error and uncertainty, related to Figure 1. A. Schematic illustration of our approach for measuring chromatic aberration. **B.** Original chromatic aberration and corrected residual error. Note, the correction is more accurate in x,y. **C**. Field portraits of the spatial organization of chromatic aberration. Each graph represents the field of view, and each arrow starts at a position of measured chromatic aberration and points in the direction of displacement between the fluorophores. The length of the arrows has been uniformly stretched to be visible on the plot. After correction, the spatially correlated nature of the aberration has been removed. **D.** Quantification of measurement error by replicate labeling. Plot shows the aggregate of all replicate labels, performed up to hundreds of imaging rounds after the original measurement to capture the greatest extent of potential sample degradation over time. **E.** Detection efficiency per SE probe across both alleles. Probes are ordered as in Table S1. A total of 14 probes were removed for low detection efficiency and/or off target reactivity and are marked in red. **F.** Number of SE alleles detected per cell before and after removing probes in E.

Figure S2, related to Figure 1

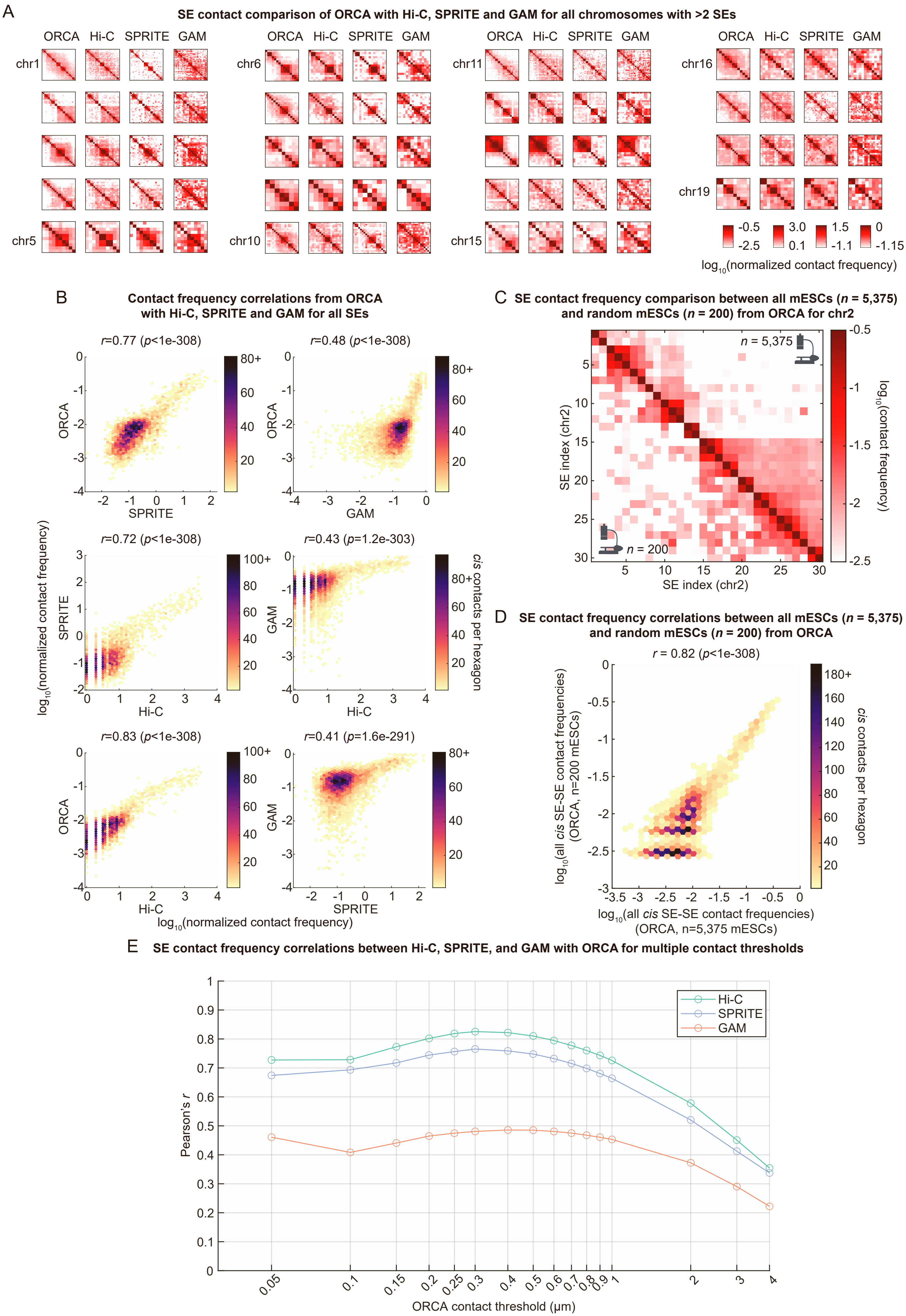

Figure S2 | Comparison of *cis* SE contacts from ORCA, Hi-C, SPRITE, and GAM, related to Figure 1. **A.** Heatmaps of the contact frequency per chromosome for ORCA, Hi-C, SPRITE, and GAM. ChrX is not shown as it contains only 2 SEs. **B.** Scatter plots comparing the normalized *cis* SE-SE contact frequencies between all 4 methods. The Pearson's correlation coefficient, *r*, and *p*-value for the $\log_{10}$(normalized contact frequencies) are shown above each plot. **C.** Heatmap comparing the ORCA contact frequency of SEs on chromosome 2 between all mESCs (*n*=5,375 cells) and a random subset of mESCs (*n*=200 cells), using a contact threshold of 300 nm. **D.** Scatter plot comparing all *cis* SE contact frequencies measured by ORCA between all mESCs (*n*=5,375 cells) and a random subset of mESCs (*n*=200 cells), using a contact threshold of 300 nm. Pearson's *r* and *p*-value for the $\log_{10}$(contact frequencies) are shown above the plot. **E.** Pearson's correlation of $\log_{10}$(*cis* SE contact frequencies) between Hi-C, SPRITE, GAM and multiple contact thresholds for ORCA. The x-axis is $\log_2$ scaled.

Figure S3, related to Figure 2

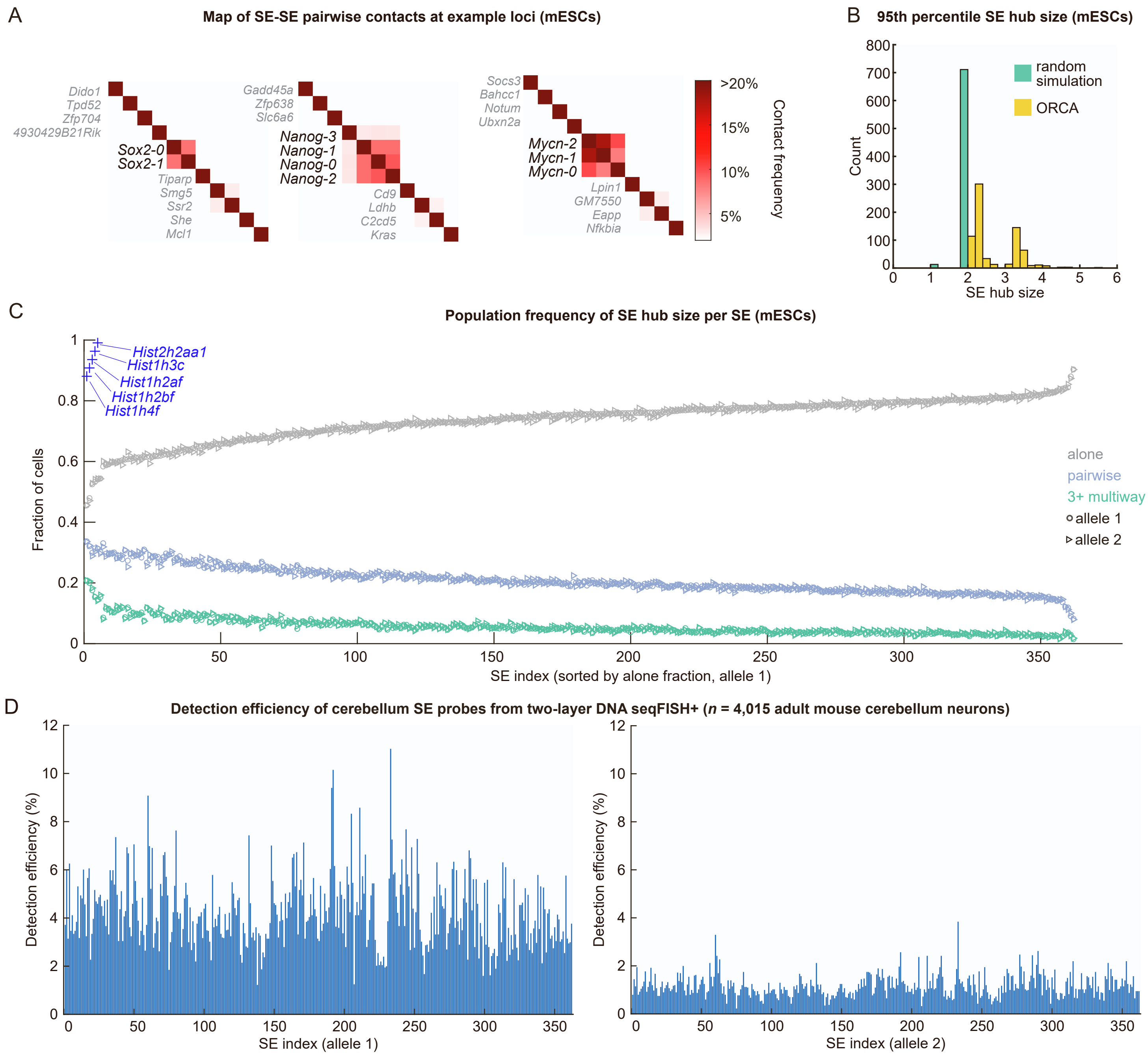

Figure S3 | Additional validation and characterization of SE interactions, related to Figure 2. A. High-frequency contacts for SEs at *Sox2*, *Nanog*, and *Mycn*. Suffixed numbers indicate SEs that were assigned to the same gene or are known to interact; a '0' indicates an SE that is at the promoter. **B.** 95th percentile SE hub size for all SEs detected by ORCA (yellow) and 724 randomly positioned SEs (green). **C**. The cell fraction for each SE when it is alone, in pairwise, or in multiway (3+) hubs. SEs are rank-sorted by the alone cell fraction size of allele 1. The five SEs with the lowest alone fraction size are indicated with a blue '+' and named. **D.** Detection efficiency of two-layer DNA seqFISH+ probes for each cerebellum SE in adult mouse cerebellum neurons, separated between both alleles.

Figure S4, related to Figure 3

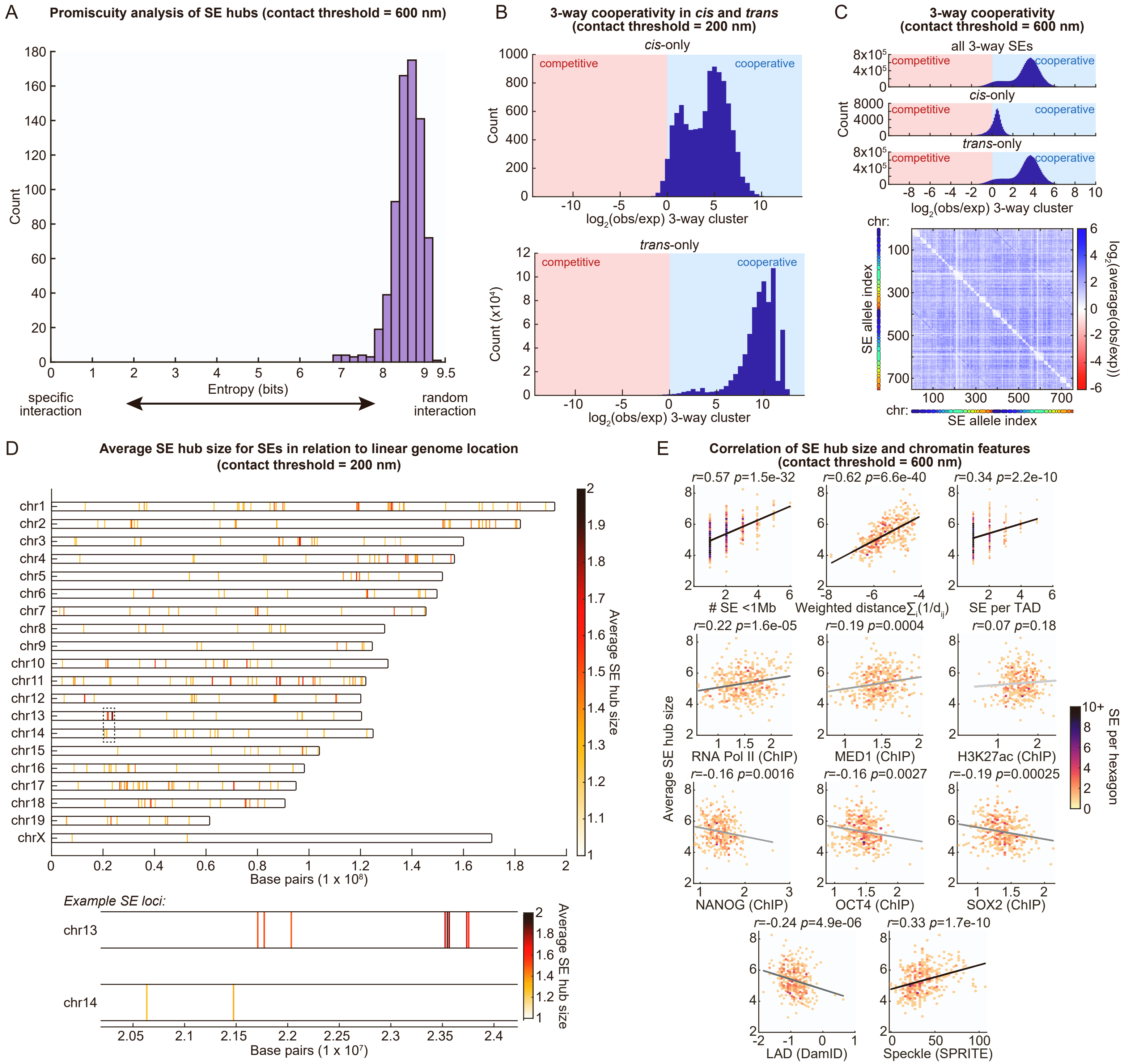

Figure S4 | Additional promiscuity, cooperativity, and correlation analyses, related to Figure 3. A. Computation of Shannon entropy for each SE over all SE hub interactions, using a contact threshold of 600 nm. **B**. Cooperativity analysis of all 3-way SE triplets ($\log_2$(O/E)), using a contact threshold of 200 nm and sorted into *cis*- and *trans*-only interactions. **C**. Cooperativity analysis of all 3-way SE triplets ($\log_2$(O/E)) using a contact threshold of 600 nm. Heatmap shows the average cooperativity score across all possible third SEs for each SE triplet combination. **D.** Chromosome maps showing the linear genomic locations for all SEs measured by ORCA and colored by average SE hub size using a contact threshold of 200 nm. Due to the large linear size of chromosomes, multiple SEs overlap each other as illustrated by the zoom-in of SEs on chr13 and chr14 (bottom). **E**. Scatter plots comparing common SE features and the average SE hub size for each SE, using a contact threshold of 600 nm. Pearson's *r* and *p*-value are above each plot. Gray/black lines are linear fits to the data, and darker line colors reflect smaller *p*-values from the Pearson correlation.

Figure S5, related to Figure 4

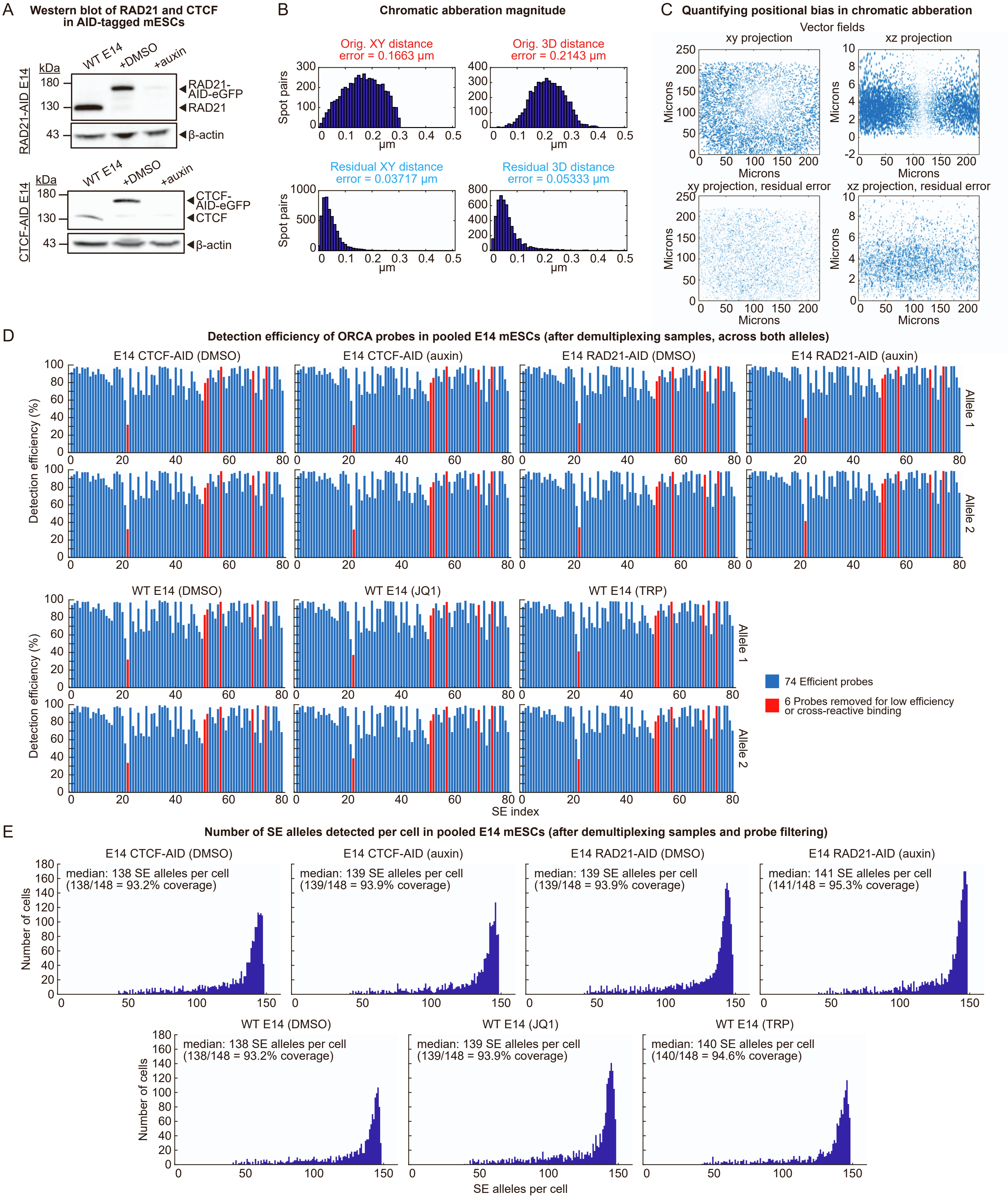

Figure S5 | Validation of perturbations on SE clustering, related to Figure 4. **A**. Western blot to visualize auxin-mediated degradation of RAD21 (top) and CTCF protein (bottom) in RAD21-AID-eGFP and CTCF-AID-eGFP E14 mESCs. Beta-actin was detected for loading control in both blots. **B.** Original chromatic aberration and corrected residual error. **C**. Field portraits of the spatial organization of chromatic aberration. Each arrow starts at a position of measured chromatic aberration and points in the direction of displacement between the fluorophores, and the length of the arrows has been uniformly stretched to be visible on the plot. **D**. The detection efficiency for each SE probe, for all perturbation and control treatments that were pooled for ORCA. **E.** The number of SE alleles detected per cell after removing selected probes, for all perturbation and control treatments that were pooled for ORCA.

Figure S6, related to Figure 4

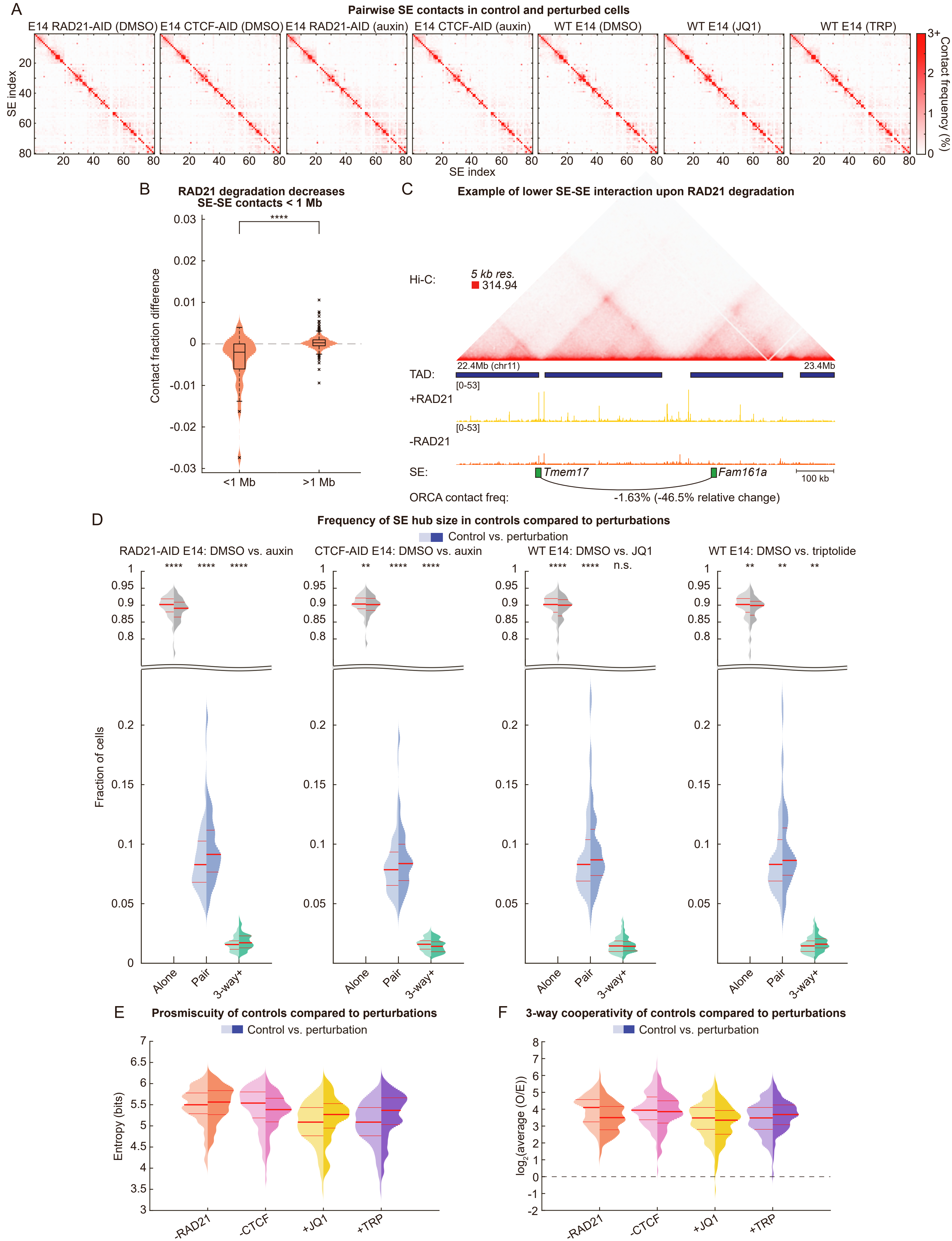

Figure S6 | Quantification of perturbations on SE clustering, related to Figure 4. **A**. Pairwise contact frequency heatmaps in perturbed and control cells for a subset of all SEs, focused on SEs from chrs 2, 3, 6, and 11 (threshold = 200 nm). **B.** Change in pairwise SE contacts binned on < 1 Mb or > 1 Mb genomic separation upon RAD21 degradation. The boxplots show the first quartile, median, and third quartile, and whiskers are 1.5 times the interquartile range. Outliers are indicated by an x. (****, p < 0.0001; Wilcoxon rank sum test). **C.** Change in contacts between SEs assigned to *Tmem17* and *Fam161a* in context of mESC Hi-C (Bonev et al. 2017 [S1]), mESC TADs (Bonev et al. 2017 [S1]) and mESC RAD21 ChIP upon AID-mediated degradation (Hsieh et al. 2022 [S2]). The change in the absolute and relative contact frequencies upon RAD21 degradation, measured by ORCA, is shown below the arc. **D**. The cell fraction for each SE when it is alone, in pairwise, or in multiway (3+) hubs in control and perturbed cells. (**, p < 0.01; ****, p < 0.0001; n.s., non-significant; Wilcoxon signed rank test). **E**. The entropy score for SE alleles in control and perturbed cells. **F.** The average 3-way cooperativity score for SE alleles in control and perturbed cells. **D-F.** The middle line represents the median, and the upper and lower lines represent the 75th and 25th percentiles, respectively.

Figure S7, related to Figure 5

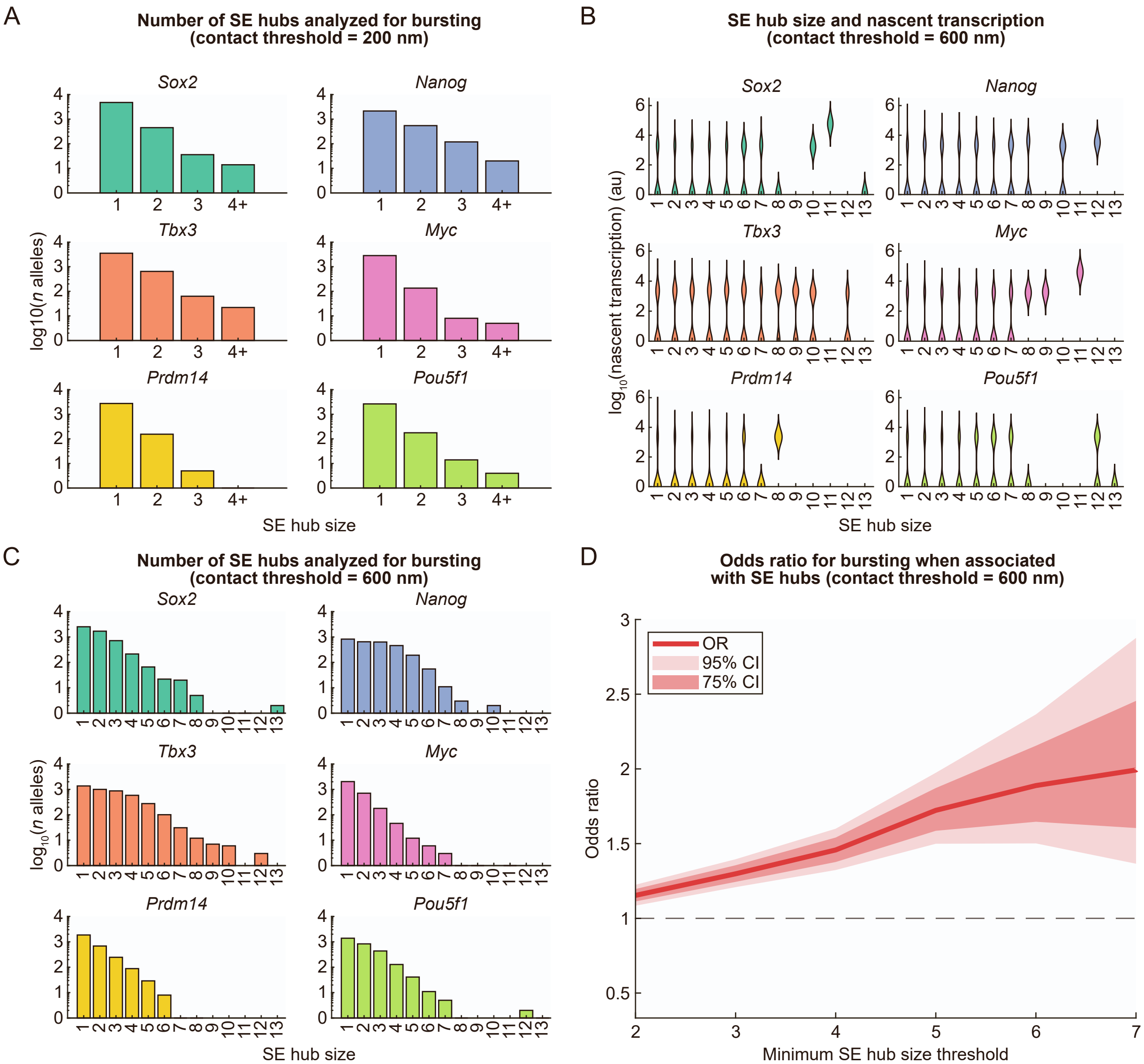

Figure S7 | Relation between transcriptional bursting and SE hubs, related to Figure 5. A. Number of alleles for each SE hub size, calculated with a 200 nm contact threshold. **B**. Distributions of nascent transcription intensity in arbitrary units (au) of the six measured genes for increasing SE hub sizes, calculated with a 600 nm contact threshold across *n*=1,727 cells. **C**. Number of alleles for each SE hub size, calculated with a 600 nm contact threshold. **D**. Odds ratio of active transcription across all of the six genes given the indicated minimum SE hub size, calculated with a 600 nm contact threshold. Shaded areas indicate the 75% and 95% confidence intervals. OR, odds ratio; CI, confidence interval.

Supplementary References

[S1] Bonev, B., Mendelson Cohen, N., Szabo, Q., Fritsch, L., Papadopoulos, G.L., Lubling, Y., Xu, X., Lv, X., Hugnot, J.-P., Tanay, A., et al. (2017). Multiscale 3D Genome Rewiring during Mouse Neural Development. Cell *171*, 557–572.e24.

[S2] Hsieh, T.-H.S., Cattoglio, C., Slobodyanyuk, E., Hansen, A.S., Darzacq, X., and Tjian, R. (2022). Enhancer-promoter interactions and transcription are largely maintained upon acute loss of CTCF, cohesin, WAPL or YY1. Nat. Genet. *54*, 1919–1932.
