# Supplementary material for: Single-cell 3D genome imaging shows super-enhancer hubs are rare, nonspecific, and neither necessary nor sufficient for transcriptional bursting
Source: Cell Genom. 2026 Aug 17;6(10):101331. doi: 10.1016/j.xgen.2026.101331 (PMC13689515; doi:10.1016/j.xgen.2026.101331)
Supplement: Supplementary file 2 [file mmc6.pdf]

Cell Genomics

Article

Single-cell 3D genome imaging shows super-enhancer hubs are rare, nonspecific, and neither necessary nor sufficient for transcriptional bursting

Graphical abstract

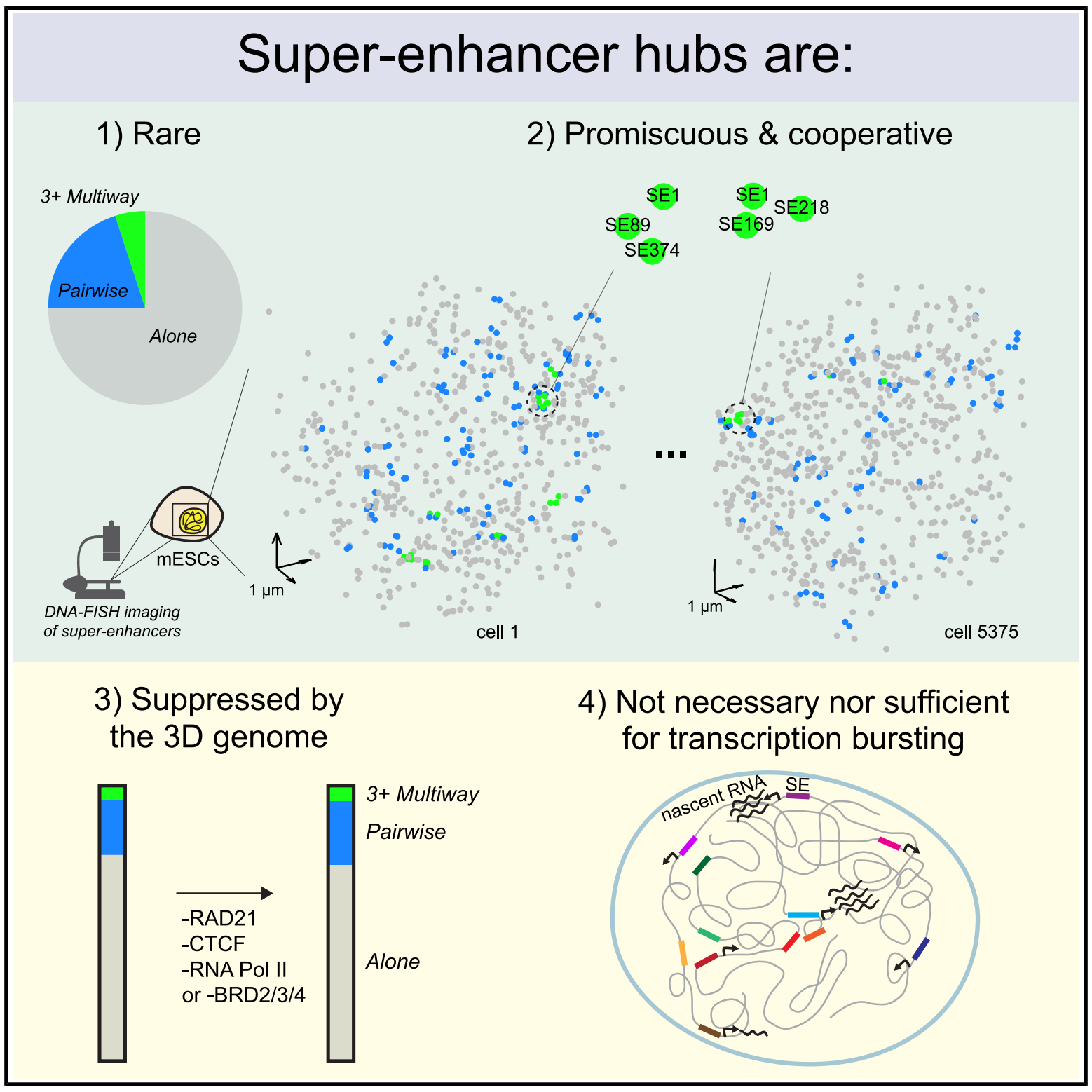

Highlights

- Super-enhancer hubs are rare in single cells
- Rare hubs are promiscuous, cooperative aggregates
- Frequency of clustering increases upon perturbation of 3D genome organization
- Super-enhancer hubs are neither necessary nor sufficient for transcription bursts

Authors

Derek J. Le, Antonina Hafner, Achuthan Raja Venkatesh, ..., Ana Novacic, Kevin C. Wang, Alistair N. Boettiger

Correspondence

boettiger@stanford.edu

In brief

Super-enhancers have been proposed to cluster in the nucleus when compared to other genomic elements. Le et al. found that super-enhancer clustering was rare and driven by non-specific interactions. Perturbing regulators of genome organization increased these interactions, suggesting they may represent tolerated cellular errors that contribute to transcriptional crosstalk.

Le et al., 2026, Cell Genomics *6*, 101331
October 14, 2026 © 2026 The Authors. Published by Elsevier Inc.
https://doi.org/10.1016/j.xgen.2026.101331

CellPress

Cell Genomics

CellPress
OPEN ACCESS

Article

Single-cell 3D genome imaging shows super-enhancer hubs are rare, nonspecific, and neither necessary nor sufficient for transcriptional bursting

Derek J. Le,[1,2,3,6] Antonina Hafner,[1,6,7] Achuthan Raja Venkatesh,[1,4] Tee Udomlumleart,[1,5] Sadhana Gaddam,[3] Ana Novacic,[5] Kevin C. Wang,[2,3] and Alistair N. Boettiger[1,8,*]
[1]Department of Developmental Biology, Stanford University, Stanford, CA 94305, USA
[2]Cancer Biology Program, Stanford University, Stanford, CA 94305, USA
[3]Department of Dermatology, Stanford University, Stanford, CA 94305, USA
[4]Biophysics Program, Stanford University, Stanford, CA 94305, USA
[5]Department of Genetics, Stanford University, Stanford, CA 94305, USA
[6]These authors contributed equally
[7]Present address: Department of Discovery Oncology, Genentech, South San Francisco, CA 94080, USA
[8]Lead contact
*Correspondence: boettiger@stanford.edu
https://doi.org/10.1016/j.xgen.2026.101331

SUMMARY

Super-enhancer (SE) hubs have been proposed to coordinate gene expression through 3D genome organization and transcriptional condensates. Using multiplexed imaging, we mapped hundreds of SEs in thousands of mouse embryonic stem cells and also paired SE position with nascent transcription measurements. We found that most SEs are spatially isolated, with multiway SE hubs occurring in only a small fraction of cells. Rare hubs were largely promiscuous, cooperative aggregates shaped by genomic proximity, nuclear speckle association, and general transcription machinery occupancy. Perturbing cohesin, CTCF, BET proteins, or RNA polymerase II showed that normal genome organization generally suppresses SE clustering. Combined RNA and DNA imaging demonstrated that SE hubs were neither necessary nor sufficient for transcriptional bursting, although larger hubs weakly increased burst probability. These results challenge models in which SE hubs are a dominant mechanism of enhancer function and instead suggest rare transcriptional crosstalk.

INTRODUCTION

Enhancers play a pivotal role in transcriptional control in higher eukaryotes, binding cell-type-specific transcription factors and activating distal cognate promoters to provide the context-specific gene expression needed for development and for responses to changes in environmental signals.[1] Enhancer-mediated gene activation depends on proximity between the enhancer and target promoter, which is constrained by the 3D organization of the genome. Traditional models suggest that such regulatory interactions generally occur in a pairwise manner, in which individual enhancers contact individual genes.[2,3] However, recent studies have suggested that some enhancer-based regulation involves multi-enhancer contacts, including interactions from extremely distal enhancers (>10 Mb) and even *trans* contacts (for review, see Mir et al.,[4] Lim and Levine,[5] Galouzis and Furlong,[6] Hamamoto and Fukaya,[7] Nollmann et al.,[8] and Zhao and Faryabi[9]). This hub model challenges the conventional understanding of enhancers as largely pairwise and largely locally regulated.

Interest in hub-mediated regulation has spurred the development of new technologies to detect multiway interactions. For example, Tri-C,[10] TM3C,[11] COLA,[12] C-Walks,[13] MC-4C,[14] Pore-C,[15] triplet-Hi-C,[16] GAM,[17] and SPRITE[18] have all reported preferential 3-way interactions among enhancers. Among these clustered enhancers, the large, highly occupied enhancers, sometimes termed super-enhancers (SEs),[19–21] are most enriched.[10,14–18] The term SE was first introduced in mouse embryonic stem cells (mESCs) to define a superlative group of enhancers with disproportionately high levels of transcription factors in concentrated genomic windows of a few to a few tens of kilobases, which tended to be near genes with important roles in cell identity.[19–21] Despite this growing evidence that enhancers cluster into hubs more often than other types of DNA, it is currently unclear at what absolute frequency clustering occurs. Relative frequencies (as measured by prior techniques) can tell us that SE hubs are 2-fold more common than hubs of non-SE chromatin; however, the biological implications are different if the absolute frequency of clustering is observed in 1% vs. 0.5% of cells compared to 40% vs. 20% of cells. Sequencing-based approaches to measuring clustering must

This is an open access article under the CC BY license (http://creativecommons.org/licenses/by/4.0/).

also contend with recovery bias and amplification bias,[22] which may skew detection enrichment. Technical parameters, such as crosslinking time, fragment length, or slice size, further constrain the analysis of elements in a cluster, with statistical power additionally confined by the number of cells and the recovery rate of the multiway interactions.[23]

Multiway enhancer interactions have been hypothesized to have synergistic, additive, competitive, and even redundant effects on gene expression, but the precise relationship between multiway enhancer clustering and transcription remains unsolved, awaiting measurement of both multiway contacts and expression in the same single cell.[24] This gap has spurred development of new multimodal single-cell omics technologies, including methods that can find multiway contacts in single cells, such as scSPRITE,[25] and various scHi-C methods[26–28]; methods to measure pairwise DNA contacts along with RNA expression, such as HiRES[29] and GAGE-Seq[30]; and even methods to measure multiway contacts and RNA in the same cells, such as R&D SPRITE,[31] MUSIC,[32] DNA-seqFISH+,[33–35] and DNA-MERFISH.[36] However, these emerging methods currently lack the sensitivity and coverage to explore associations between enhancer hubs and transcription bursts. Concurrently, protein clusters have been seen to associate with transcriptional bursts in fixed[21] and live[37,38] cells, though it is unclear if these clusters involve enhancer hubs or individual loci. Recent reviews have especially highlighted the important gaps left in recent evidence of transcription hubs in testing what impact clustering has on transcription of associated genes.[4–8]

To overcome these constraints and better quantify SE clustering, we employed DNA fluorescent *in situ* hybridization (FISH) using optical reconstruction of chromatin architecture (ORCA) to map the 3D positions of all SEs in thousands of single cells. We combined these data with simultaneous DNA and nascent transcription labeling and also examined the effect of perturbations that impact genome organization on clustering. We found SE clusters to be rare, nonspecific, and opposed by healthy genome functions. We thus propose that SE clustering is more likely to contribute to a form of rare transcriptional crosstalk than to be a fundamental mechanism by which SEs coordinate transcription.

RESULTS

Global spatial organization of SEs in single cells

To elucidate the spatial characteristics of SE clustering, we conducted a comprehensive mapping of the 3D spatial positions of all SEs (Figure 1A). We combined the list of all SEs from the seminal work on SEs in mESCs by Whyte et al.,[19] with an additional 145 elements elevated as SEs in a later classification by Sabari et al.,[21] resulting in a master list of 374 unique SEs (Table S1). We note that several of the previously defined SEs span transcription start sites (TSSs), and thus both enhancers and some promoters are included in our assay. To precisely locate the positions of these SEs at the single-cell level, we targeted each SE with a unique sequence tag by *in situ* hybridization and detected these tags with independent fluorescent oligos in independent images using ORCA.[39] This approach is more laborious than DNA-MERFISH and related methods,[33,34,36] which assay many targets using distinct combinations of a small number of tags and require exponentially fewer imaging rounds. However, our use of unique tags allowed spatially clustered signals to be more reliably detected and identified, which is less feasible with combinatorial approaches. Imaging probes were designed by selecting 15 kb regions centered on the previously identified SE calls (Figure 1B; Table S1), ensuring uniformity in our experimental design and detection efficiency. When accounting for homologous chromosomes, these probes allowed us to discern up to 748 distinct SE alleles per cell (Figure 1C).

We imaged thousands of mESCs in hundreds of sequential imaging rounds, including control images used to quantify measurement uncertainty and correct for chromatic aberration. Following corrections for technical errors including stage drift, sample drift, and chromatic aberrations, we achieved high spatial precision, with a residual error of ~100 nm (Figures S1A–S1D). During image processing, 12 SEs were dropped from the analysis due to limited detection efficiency or off-target binding, while the remaining 362 SEs exhibited largely uniform and highly efficient detection (median >90% detection efficiency per SE probe and a median detection of 678/724 SE alleles per cell [93.6%]), resulting in high-confidence 3D localization of 724 SE alleles across 5,375 mESCs (Figures S1E and S1F). Overall, our comprehensive imaging experiment measured over 2 billion inter-SE distances across 343,000 images and 6 TB of data, providing a rich foundation for further exploration of the intricate landscape of SE-SE interactions.

To validate our ORCA dataset, we computed pairwise SE interaction maps for each chromosome (in *cis*) and conducted a thorough comparison between ORCA and three previously employed genome-wide datasets: Hi-C,[41] GAM,[17] and SPRITE,[18] each also collected in mESCs. Hi-C involves *in situ* ligation of restriction-digested genomes to identify 3D proximity among sequence elements. GAM utilizes whole-genome sequencing of isolated nuclear cross-sections to reveal patterns of sequence clustering, avoiding the need for restriction digestion or proximity ligation. SPRITE employs a split-and-pool barcoding strategy to identify crosslinked sequences within the same nuclear fragment, also avoiding the use of proximity ligation. All three methods offer a relative measure of interaction frequencies across the entire SE interactome examined in our data, while differences between the methods offer a measure of control to account for method-specific artifacts or biases. There was a statistically significant correlation between the SE ORCA data and all three sequencing-based methods (Figures 1D, S2A, and S2B). Notably, all three methods exhibited a stronger correlation with ORCA than with each other (Figure 1E). Assuming that shared features among the four techniques reflect the true biology of mESC SE interactions and that divergent features indicate technique-specific artifacts, this comparison suggests that ORCA exhibits the fewest artifacts, underscoring its reliability in capturing mESC SE interactions. Technical factors, including differences in sampling depth and variability in the effective contact radius, likely contribute to residual differences between the datasets, as suggested by random sampling of a subset of cells from ORCA and also by varying the contact threshold in comparisons (Figures S2C–S2E).

CellPress
OPEN ACCESS

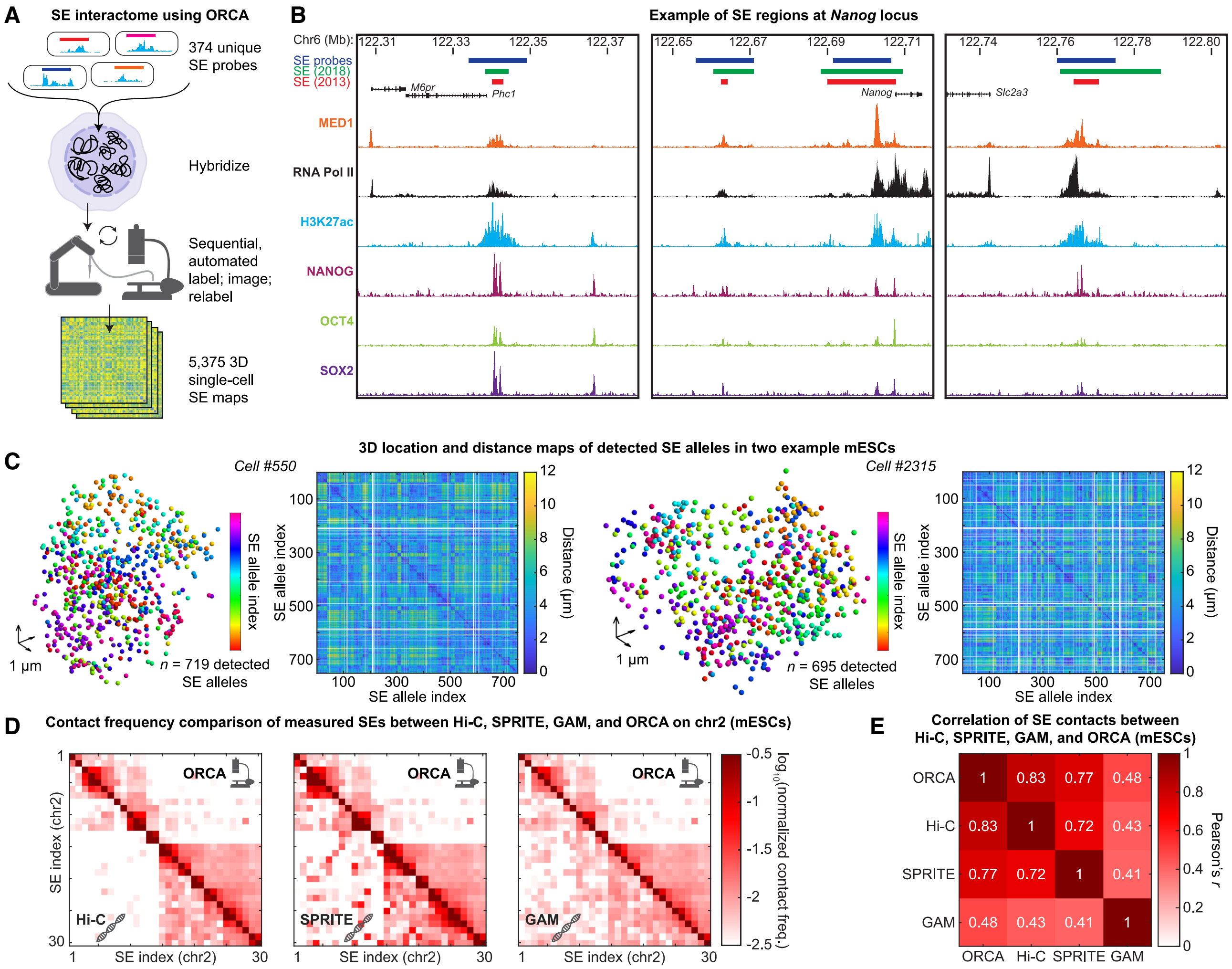

	ORCA	Hi-C	SPRITE	GAM
ORCA	1	0.83	0.77	0.48
Hi-C	0.83	1	0.72	0.43
SPRITE	0.77	0.72	1	0.41
GAM	0.48	0.43	0.41	1

Figure 1. ORCA enables visualization of all mESC SEs across single cells

(A) Schematic of approach, where each SE is individually labeled and imaged. *n* = 5,375 mESCs were imaged.

(B) UCSC Genome Browser tracks of *Nanog*-associated SEs. SE annotations by Whyte et al.[19] and Sabari et al.[21] are shown in red and green boxes, respectively. 15-kb regions, centered on Whyte et al. annotations, were picked for ORCA and are shown as blue boxes. ChIP-seq tracks of MED1, RNA Pol II,[21] H3K27ac,[40] NANOG, OCT4, and SOX2[19] were aligned to SE annotations.

(C) Examples of two mESCs showing the 3D positions of detected SE alleles and the corresponding single-cell SE-SE pairwise distance maps. White lines in the distance maps indicate SE alleles that were not detected within the respective cell.

(D) Heatmaps comparing the relative contact frequencies among SEs on chromosome 2 (all other chromosomes are shown in Figure S2A) measured by ORCA (<300-nm threshold), Hi-C,[41] SPRITE,[18] and GAM[17] in mESCs.

(E) Pearson correlation of $\log_{10}$(contact frequencies) for *cis* SE contacts across all chromosomes between ORCA, Hi-C, SPRITE, and GAM in mESCs.

SE hubs are rare and involve few SEs

To determine what distance threshold to use to identify SE interactions, it is instructive to know the physical size of a typical SE and thus understand what center-to-center distances between SEs are consistent with contact. The well-studied *Sox2* control region (SCR) SE has been previously imaged with probes tiling the ∼15-kb SE region in 5 kb steps.[39,42] We found that the median distance between the two adjacent probes was ∼175 nm, while probes at opposite ends of the single SE were ∼225 nm apart in our measurements and in independent results from Huang et al.[42] (Figure 2A). Based on these measurements, we decided to use 200 nm as a cutoff distance for calling SE contacts.

At this threshold, SE-SE interactions of known regulatory importance[25,42–45] exhibited contact frequencies of ∼10%, as expected from prior work[39,42] (Figure S3A). By contrast, contacts among nearly all other SE pairs were rare, occurring in <1% of alleles (Figures 2B–2D). To further analyze SE clustering, we leveraged our single-cell data to compute the fraction of cells in which each SE allele was in contact with any of the other 723 alleles in its nucleus (Figure 2E). We found that nearly all SE alleles were alone in 70%–80% of cells (Figure 2E). Different SEs formed hubs of 3 or more in only 4%–7% of cells (lower and upper quartiles over SEs) (Figure 2E). This low frequency of SE hubs corresponded to an average cluster size of 1.2–1.9 SEs per hub,

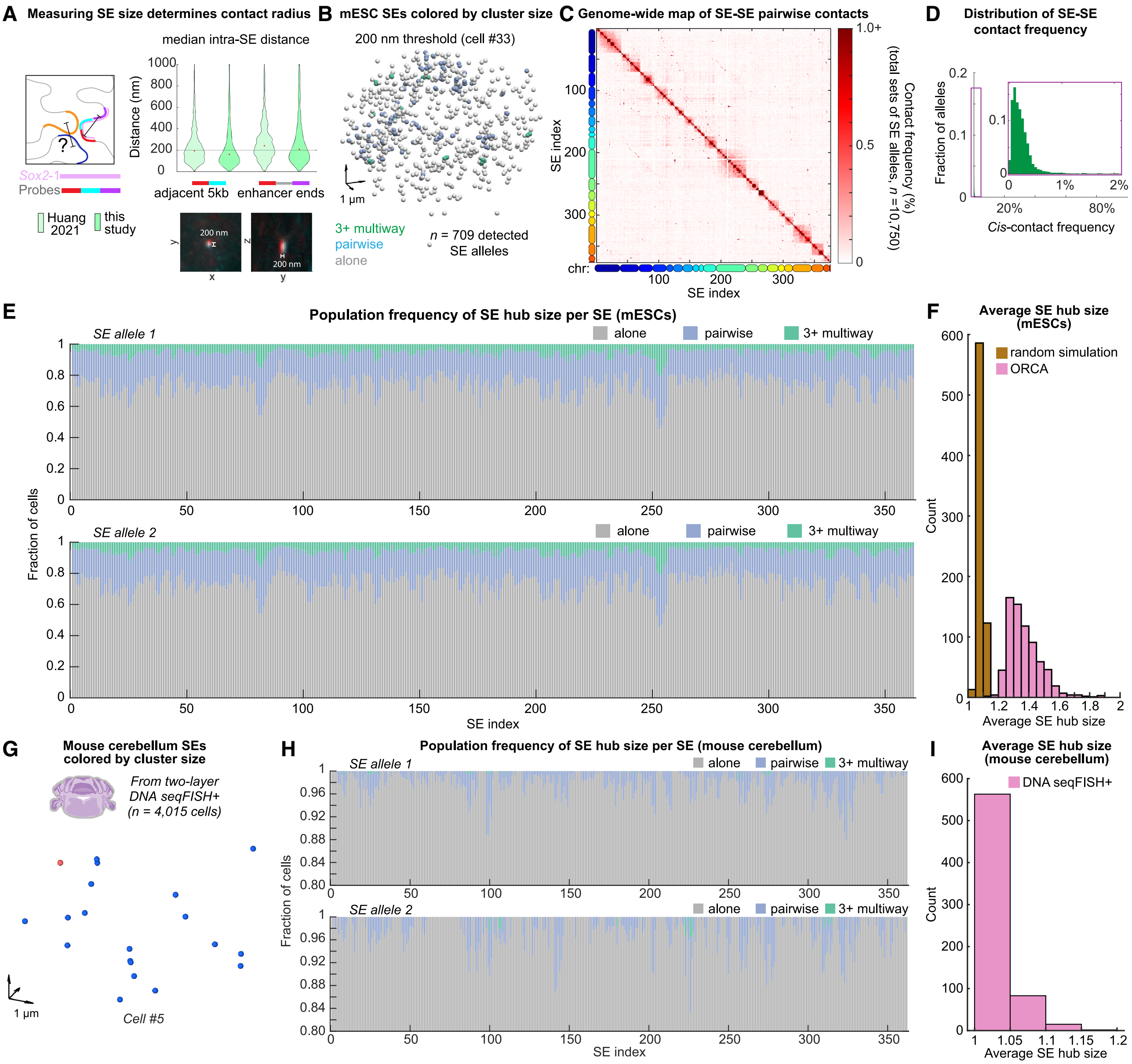

Figure 2. Multiway SE contacts are infrequent

(A) Schematic illustrating the approach of determining the distance threshold for SE-SE contact. Colored bars show the 15-kb *Sox2* SCR SE (*Sox2*-1) tiled with 3 adjacent 5-kb probes. Quantification of the interprobe distance of adjacent and end-to-end pairs by Huang et al.[42] and this study: *n* = 430, *n* = 2,359, *n* = 479, and *n* = 1,312.

(B) Example of a single cell with 3D positions of all imaged SEs colored by the hub size (number of other SEs within 200 nm).

(C) Pairwise heatmap of all SE contacts detected by ORCA.

(D) Distribution of contact frequency for all *cis* SE-SE pairs.

(E) The cell fraction for each SE when it is alone, in pairwise, or in multiway (3+) hubs. SEs are ordered by genomic location and separated between both alleles.

(F) Average SE hub size for all SEs detected by ORCA (pink) and for simulated cells with 724 randomly positioned SEs (brown).

(G) Example of an adult mouse cerebellum neuron with the 3D positions of detected cerebellum SE-associated probes from two-layer DNA seqFISH+.[34] Blue spheres are SEs assigned to allele 1, and red spheres are SEs assigned to allele 2.

(H) The cell fraction for each SE when it is alone, in pairwise, or in multiway (3+) hubs in cerebellum neurons. SEs are ordered by genomic location and separated across both alleles.

(I) Average SE hub size for all SEs detected by two-layer DNA seqFISH+ in cerebellum neurons.

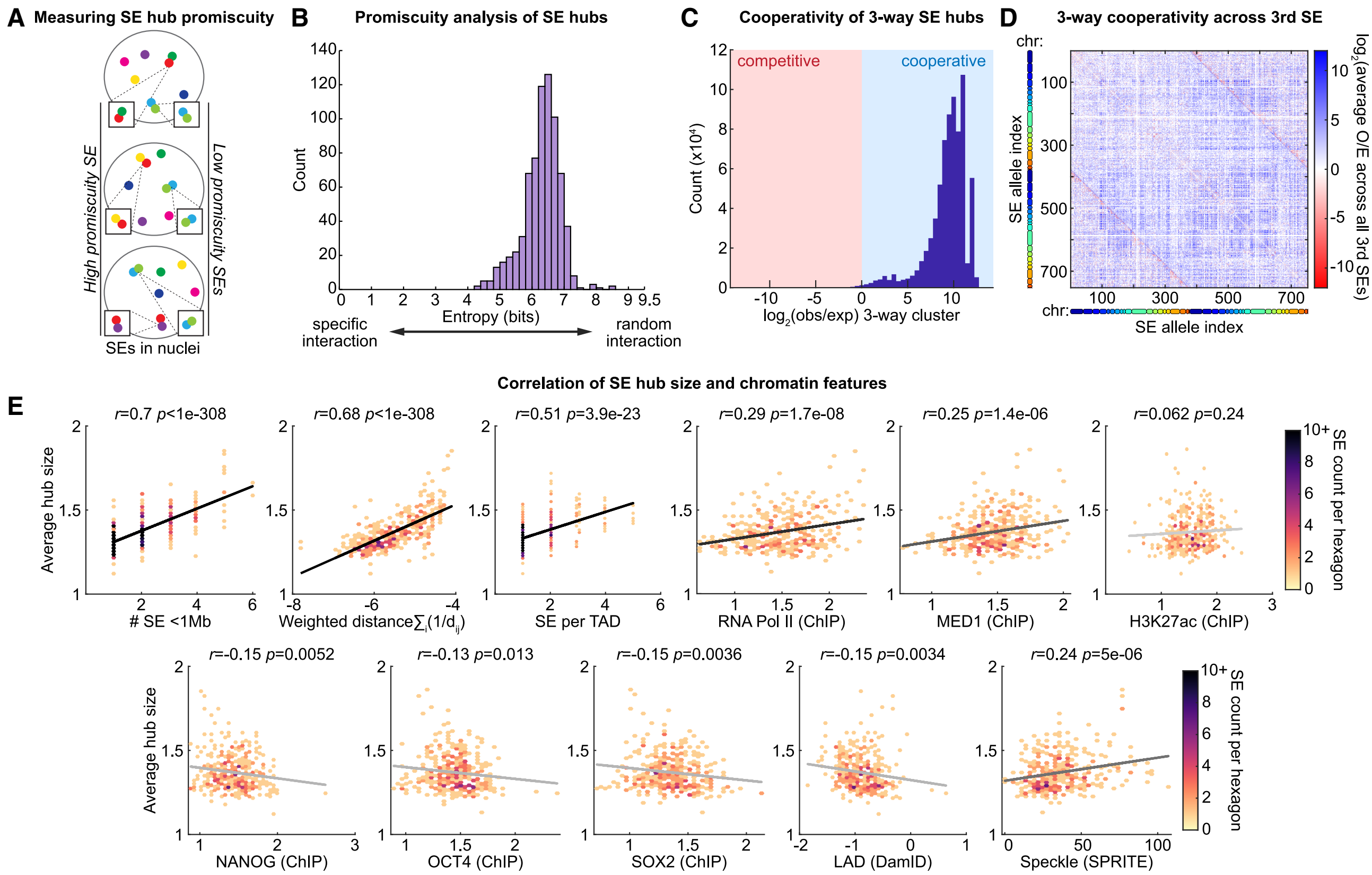

Figure 3. SE hubs are rare, nonspecific, cooperative aggregates

(A) Schematic of a high-promiscuity SE interacting with different SEs and a low-promiscuity SE interacting with a specific SE.

(B) Distribution of SE hub promiscuity as measured by Shannon entropy for each SE allele over all hub interactions. The maximum entropy score is ~9.5 bits, representing a uniformly random distribution of SEs.

(C) Cooperativity (log_2[observed/expected]) for all 3-way SE allele interactions.

(D) Heatmap of average cooperativity among SE allele triplets. For each pair of SEs, the heatmap shows the average cooperativity of all possible third SEs.

(E) Scatterplots comparing common SE features and the average SE hub size for each SE. Pearson's correlation coefficient, *r*, and *p* value are above each plot. Gray/black lines are linear fits to the data, and darker line colors reflect smaller *p* values from the Pearson correlation.

and even the top 5th percentile largest clusters for each SE were composed of only 2–4.5 SEs (Figure S3B). However, SEs were more clustered than expected had they been randomly distributed within the nuclear volume (Figure 2F). The SEs of the replication-dependent *Hist1* histone cluster genes, which were previously shown to form a high-frequency multiway hub by SPRITE,[18] stood out in our data from other SEs in their clustering propensity, forming hubs of 3+ in 20% of cells (Figure S3C). Overall, these data also clearly showed that isolation was the typical state for any given SE and that multiway contacts were rare.

While SE-SE clustering has been primarily reported in stem cells, it is possible that clustering is substantially more common in more differentiated cell types. To test this hypothesis, we reanalyzed recent two-layer DNA seqFISH+ data from Takei et al.[34] from adult mouse cerebella. We first defined a set of 362 SEs using H3K27ac chromatin immunoprecipitation using sequencing (ChIP-seq) data from mouse cerebella[46] and then quantified interactions of these regions in single neurons using the multiplexed FISH data (Figure 2G; Table S2). We observed that SEs were also generally distributed throughout the nuclei, with few pairwise contacts or 3-way interactions (Figures 2H and 2I). While the sparser nature of the two-layer combinatorial barcoding precluded a direct, quantitative comparison, these data indicated that SEs in the post-mitotic brain were mostly isolated, consistent with our focused analysis in stem cells (Figure S3D).

Most SE hubs are nonspecific, cooperative aggregates

We next asked if rare SE hubs reflected a generic tendency to cluster or a more complex layer of specificity and organization (Figure 3A). To measure the specificity of SE hubs, we performed a promiscuity analysis by calculating the Shannon entropy score for each SE (see STAR Methods). An entropy value of 0 indicates that an SE interacted with the same specific set of SEs across all cells, while an entropy value of 9.5 indicates fully random interactions at equal frequency with any of the 723 other SEs in the cell ($log_2(724) \sim 9.5$). The entropy value of most SEs was 5.5–7 (>8 at 600-nm contact threshold; see Figure S4A), indicating that most SEs are promiscuous (Figure 3B). The *Hist1* gene

cluster-associated SEs stood out as the least promiscuous, while a few SEs, such as *Rbfox2*, were unusually promiscuous.

Since promiscuity analysis indicated that SE hubs were mostly nonspecific, we then asked if rare SE hubs aggregated cooperatively, similar to the relatively nonspecific and promiscuous cooperative assembly of biomolecular condensates.[47,48] In cooperative assembly, a larger aggregate is more likely to recruit an additional member than a small aggregate. For all possible SE triplets, we quantified cooperativity by computing the observed frequency of a particular triplet and dividing this by the expected frequency based on the observed pairwise interaction frequency (see STAR Methods). Across all 3-way combinations, we found that SE hubs were almost always cooperative, with most 3-way SE hubs occurring between 2 and 2^{12} times more often than expected based on the observed pairwise contact frequencies (Figure 3C). Restricting the analysis to *cis*-only SEs showed similar cooperativity (Figure S4B). We then computed a map of cooperativity by plotting how cooperative or competitive an SE is on average, as a function of its partners. Most SE pairs were cooperative (blue), with little systematic spatial structure, indicating that there is little specificity in the recruitment of a third SE as a function of the identity of the original pair (Figure 3D). Cooperativity for 3-way hubs was also observed at contact thresholds of 600 nm, indicating this observation is not sensitive to the restrictive threshold (Figure S4C).

We next wanted to understand if there were any SE characteristics that correlated with hub size. We considered the genomic position of SEs (due to the properties of the DNA fiber), transcription factor and general transcription machinery occupancy (due to their contribution to cooperative biomolecular condensate formation), and lamin-associated domain (LAD) or nuclear speckle association (due to their nuclear subcompartmentalization) (Figure 3E). We found that SE hub size correlated with SE linear genomic density (SEs/1 Mb), number of SEs per topologically associating domain (TAD), and weighted *cis* inter-SE linear genomic distance (Figures 3E and S4D). MED1 occupancy, RNA Pol II occupancy, and nuclear speckle association also showed statistically significant but small correlations with hub size, while other factors, including H3K27ac, did not (Figure 3E). Contact thresholds of 600 nm produced similar correlations with cluster size (Figure S4E). These data show that linear genomic separation is the strongest but not the only contributor to SE clustering, with other spatial features such as speckle association and RNA Pol II occupancy making mild contributions.

Endogenous genome forces oppose SE hub formation

To further explore the effect of genome structure on SE hub formation, we measured SE 3D organization following several treatments that are potentially disruptive to SE organization in mESCs, focusing on a selection of 74 SEs due to the experimental complexity (Figure 4A; Table S3). To perturb 3D DNA structure, we used established auxin-inducible degron (AID) cell lines to deplete RAD21, a core component of the DNA loop-extruding cohesin complex, or deplete CTCF, a DNA-binding protein that restricts cohesin movement.[49] Since BRD4 and RNA Pol II are prone to aggregation and may cluster SEs through this mechanism, we also perturbed these proteins with small-molecule inhibitors, which interfere with their chromatin binding, protein stability, or both.[50,51] For these perturbation studies, we focused on imaging SEs on chromosomes 2, 3, 6, and 11 and used a multiplexed approach to pool perturbed and control cells to avoid batch effects.[49] We observed a high efficiency of protein degradation by western blot and a high SE detection efficiency after demultiplexing cells (Figures S5A–S5E).

We found that the SE interactome was largely robust against these perturbations, though systematic changes in each condition, consistent with the expected mechanisms of action, were observed upon quantitative comparison with controls (Figures 4B and S6A). For example, upon cohesin loss, the physical distance increased for proximal SEs within 1 Mb of each other, consistent with previous analyses[49] (Figures S6B and S6C).

In all perturbations compared to controls, most SEs increased the frequency with which they were in pairwise hubs (Figures 4C and S6D). Cohesin depletion (RAD21 degradation) and transcription inhibition (triptolide) also substantially increased the frequency of hubs of 3 or more SEs, though the spread was larger owing to the rarity of such hubs to begin with (Figures 4C and S6D). These data suggest that SE hubs are thus more generally suppressed by normal genome structure rather than being actively assembled by it.

Both JQ1 and triptolide treatment modestly increased the promiscuity of SE hubs with minor effects on cooperativity, relative to controls (Figures 4D, 4E, S6E, and S6F). RAD21 degradation also increased promiscuity and modestly decreased cooperativity in hub assembly, whereas CTCF degradation resulted in decreased promiscuity. Together, the changes in SE hubs upon perturbation are consistent with a model in which the endogenous genome forces of healthy cells largely oppose the formation of hubs while ensuring specificity in SE contacts.

SE hubs in rare events may lead to aberrant bursts of transcription

We next asked whether SE hubs affected the nascent transcription of SE-linked genes in mESCs. To do this, we performed single-molecule RNA FISH and ORCA in the same cells. For RNA FISH, we imaged nascent transcripts of 6 highly studied genes (*Nanog*, *Sox2*, *Myc*, *Pou5f1*, *Prdm14*, and *Tbx3*) and then sequentially performed ORCA DNA imaging for a subset of 44 SEs, located proximally to each of the 6 measured genes (Figure 5A; Table S4). Transcriptional burst size was quantified by measuring the intensity of the nascent RNA punctum. We observed many instances of nascent transcription from genes whose SEs were not concurrently in an SE hub. We also observed many instances with no detectable nascent transcription from genes whose SEs were in a hub (Figures 5B and S7A–S7C). This implies that SE hubs are neither required nor sufficient for transcriptional bursting. Nonetheless, across all genes, the odds of observing a transcription burst increased modestly but steadily as a function of the hub size of the associated SE (Figure 5C). The increase in odds of observing a transcription burst did not depend on the contact threshold used to determine SE hub sizes (Figure S7D). To further investigate bursting of SE-linked genes, we used data from recent high-throughput imaging of nascent transcripts[34] to quantify the burst frequency of 272 of

CellPress OPEN ACCESS

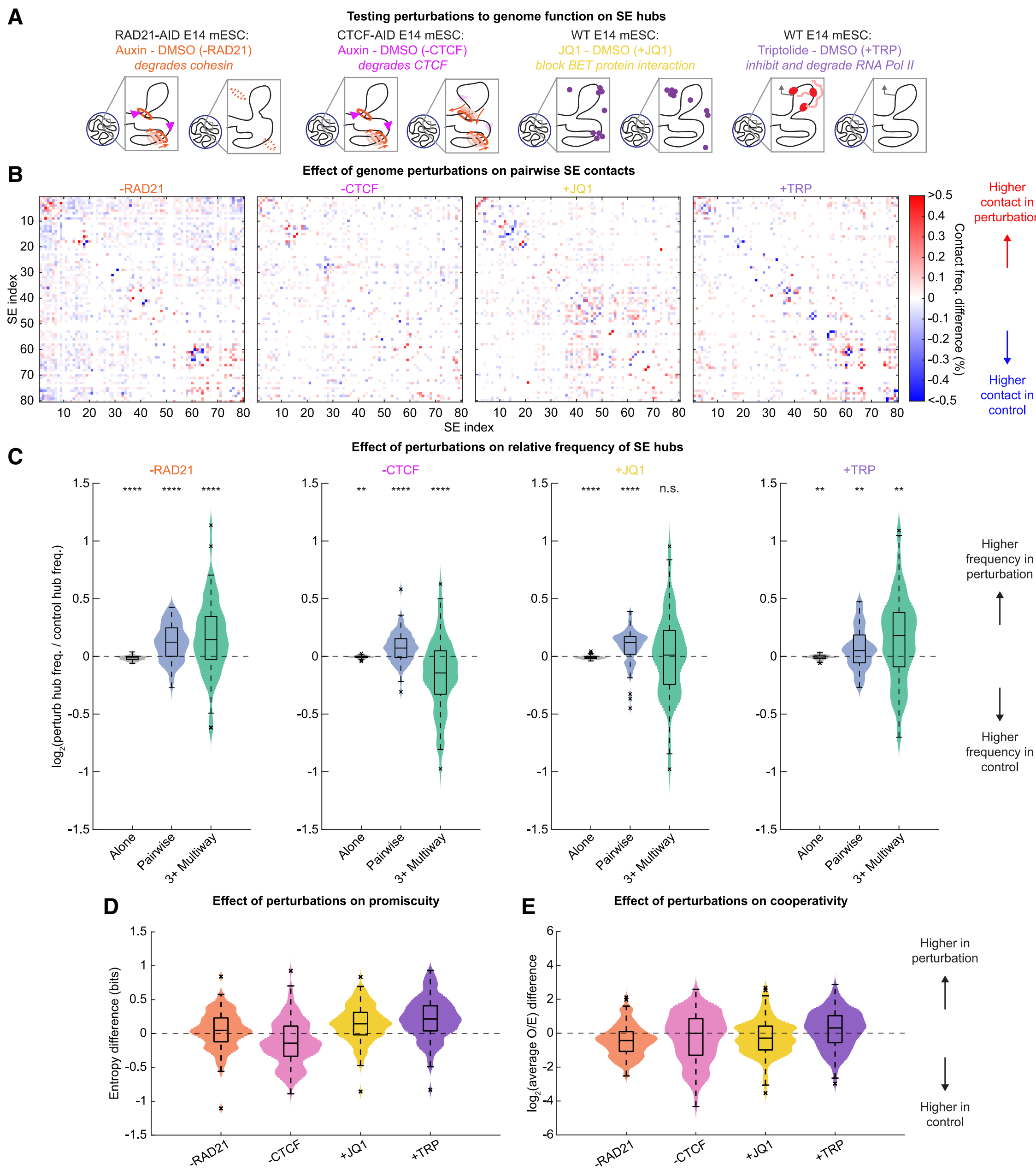

Figure 4. Perturbations to endogenous genome forces result in a minor, nonspecific increase in SE clustering

(A) The effect of each treatment performed on mESCs to perturb SE hub formation. Number of mESCs imaged per condition: RAD21-AID E14 (DMSO), $n = 2{,}108$ cells; RAD21-AID E14 (auxin), $n = 2{,}039$ cells; CTCF-AID E14 (DMSO), $n = 1{,}774$ cells; CTCF-AID E14 (auxin), $n = 1{,}680$ cells; wild-type (WT) E14 (DMSO), $n = 1{,}394$ cells; WT E14 (JQ1), $n = 1{,}892$ cells; and WT E14 (triptolide), $n = 1{,}434$ cells.

(B) Bootstrapped (90% confidence interval) pairwise contact frequency difference maps between perturbed and control cells for a subset of all SEs, focused on SEs from chromosomes 2, 3, 6, and 11.

(C) The distribution of $\log_2$ fold changes in frequency of SE hub size per SE, calculated as $\log_2$(perturb hub frequency/control hub frequency) for each SE across perturbations (**$p < 0.01$, ****$p < 0.0001$, and n.s., non-significant; Wilcoxon signed rank test).

(D) Promiscuity differences for SE alleles across perturbations.

(E) Average cooperativity differences for SE alleles across perturbations.

(C–E) The boxplots show the first quartile, median, and third quartile, and whiskers are 1.5 times the interquartile range. Outliers are indicated by an x.

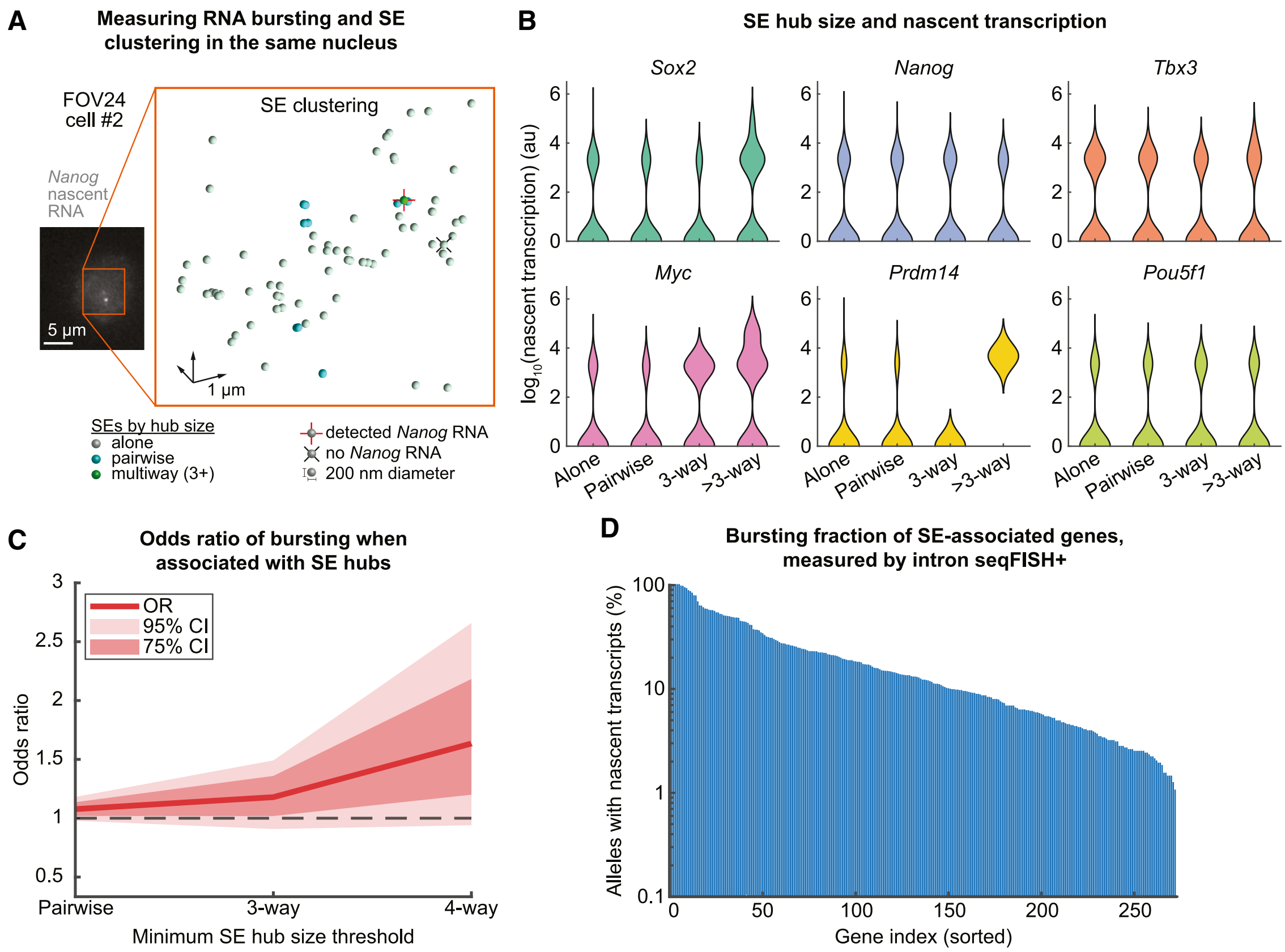

Figure 5. SE hubs are neither necessary nor sufficient for transcriptional bursting

(A) Example of a single cell with the nascent RNA for *Nanog* and the 3D positions of a subset of SEs, imaged with RNA FISH and ORCA. The color of SE spheres corresponds to SE hub size.

(B) Distributions of nascent transcription intensity in arbitrary units (a.u.) of the six measured genes for different SE hub sizes across n = 1,727 cells.

(C) Odds ratio of active transcription across all six genes given the indicated minimum SE hub size. Shaded areas indicate the 75% and 95% CIs. OR, odds ratio; CI, confidence interval.

(D) Analysis of SE alleles with detectable nascent transcription from intron seqFISH+.[34]

the 374 SE-associated genes in our study. Over half of the genes showed nascent transcript bursts for >10% of alleles in mESCs, contrasting the much lower frequency at which the SE for these genes formed hubs (Figure 5D). Given that SE hub-associated bursts accounted for such a small fraction of total bursts, we suggest that this subtle yet measurable impact on transcription is more likely to be a tolerated background crosstalk between genes and non-cognate SEs rather than a core mechanism of gene regulation.

DISCUSSION

Enhancer clustering has been invoked to play important roles in diverse molecular processes, including cell fate specification,[5,7,24,52] cancer,[52] emergent behaviors such as liquid-liquid phase separation (LLPS),[4,8,24,47] and more.[7,8] Here, we focused on stem cell SEs as a strongly supported model of enhancer clustering, using a comprehensive list of 374 SEs defined in mESCs.[10,15–18] On a single-cell level, we found hubs of SEs to be surprisingly rare in normal cells. In contrast to the frequent transcriptional bursts seen by SE-associated genes, this rarity suggests that clustering may not play a major functional role in transcription and could instead reflect an "error" in genome organization and transcription regulation.

Recent work has shown that general transcription machinery, including RNA Pol II, MED1 and BRD4, form LLPS-driven transcriptional condensates at high concentrations.[21,50,53] *In vivo*, these condensates colocalize on SEs.[21,50,53] Together, these observations offered a bold hypothesis in which SE clustering is the first step in creating a phase-separated transcriptional condensate and the emergent properties of such condensates then provide unique emergent types of transcriptional regulation. Synthetic experiments provide support for this model—clustering of synthetic enhancers in linear arrays, creating a permanent 3D clustering of binding sites, nucleated LLPS-driven condensates when the arrays crossed a critical size scale.[54] In this view, the reason SEs cluster into hubs is to raise the concentration of transcription machinery components above the critical threshold that triggers LLPS. Some features of our data actually fit predictions of this model: for example, SEs that on average have larger hubs correlate with an average higher occupancy of RNA Pol II and MED1. Nonetheless, the rarity of clustering,

compared to the relative frequency of transcription bursts or the frequency at which condensates have been reported, is not consistent with this view in which SE clustering and resulting LLPS of a transcriptional condensate is a primary mechanism of transcriptional regulation for SE-associated genes.

An alternative model to one in which clustering plays a novel and essential role in gene activation through nucleation of LLPS is one wherein rare SE hubs correspond to a background level of nonspecific aggregation. Such a model is supported by our perturbation data. Upon RAD21 degradation, the frequency of SE hubs generally increased, while contacts between known E-P pairs (<1 Mb) decreased,[49] suggesting that cohesin normally opposes ectopic hubs. Cohesin's role in mitigating SE clustering is further supported by the enriched detection of multiway SE hubs upon RAD21 degradation in human cancer cells (using bulk, population-based Hi-C)[16] and by cohesin's role in suppressing coactivation of *cis*-linked genes in mESCs.[55] Interestingly, inhibition of RNA Pol II also increased the frequency of multiway SE hubs, which could be explained by prior observations of RNA Pol II depletion resulting in the formation of new loops potentially through CTCF/cohesin- or Polycomb-dependent mechanisms.[56,57] Most perturbations also increased hub promiscuity. Together, these observations suggest that the endogenous genome organization functions to minimize background, nonspecific hub formation among SEs.

The model of SE hubs as rare background aggregates is further supported by our observations that SE hubs are neither necessary nor sufficient for transcriptional bursting. The presence of SE hubs was only marginally associated with an increase in the probability of observing transcription bursting, but given the rarity of hubs relative to the expression level of SE-associated genes, promiscuous clusters are probably too rare to play a major functional role in normal cell behavior. Supporting the conclusion that hub formation has a minimal impact on transcription, a recent sequencing-based study found little link between multiway hub frequency and transcription, despite observing a correlation between pairwise contacts and transcription.[58] Moreover, most individual cells are unlikely to experience transcriptionally productive SE hub interactions even once per cell cycle, given the large distances between SEs and the slow motion of chromatin at this length scale in the dense nucleus.[59] Consequently, SE hubs may instead contribute to a form of rare transcriptional crosstalk that is checked by cohesin, CTCF, and potentially other endogenous genome regulators.

Future investigation will be needed to fully distinguish whether SE hubs are an adaptive solution that has emerged through positive selection in evolution, a detrimental source of transcriptional noise that evolution has been unable to fully purge, or a context-dependent combination of both. Extending the framework we established here to other cell types, such as terminally differentiated cells or pathological conditions, may uncover conditions in which clustering plays a more prevalent role than observed in stem cells. Indeed, elevated degrees of enhancer clustering have been reported in several oncofusion pathologies, including BRD4-NUT,[60,61] NUP98-HOXA9,[62,63] and EWS-FLI1,[64] consistent with a picture in which clustering may be detrimental. Evidence for increased enhancer hubs in other cancers, including triple-negative breast cancer, mantle cell lymphoma, T cell acute lymphoblastic leukemia, and glioblastoma, has been reported,[65–68] further supporting the hypothesis that SE hubs are largely avoided in healthy cells. Therefore, the few SE hubs that do occur may likely represent tolerated, cellular errors that are not essential for normal functions.

Limitations of the study

A few limitations should be kept in mind when interpreting these results. The SEs selected for this study comprise all of the mESC SEs defined by Whyte et al.[19] and an additional subset of SEs defined by Sabari et al.,[21] but the criteria used to define SEs vary.[20,69] Other SE-like loci may have a higher propensity to cluster but may be uniquely defined by other genomic features such as RNA Pol II occupancy. This study was primarily limited to SEs in mESCs, and further in-depth study in other differentiated or pathological cell types, such as cancer cells, may reveal different SE clustering patterns. Investigating more genes combined with similarly high detection efficiency for both nascent transcription and DNA could better resolve the relationship between SE hubs and transcription.

Our experiments are performed in fixed cells, and fixation can potentially induce changes in cellular structures and organization. Comparison of DNA loci measured using our fixation and hybridization protocol to the same loci measured in live cells with integrated genomic tags suggests that the magnitude of these changes is small relative to the measurement uncertainty in our data.[70] DNA denaturation and hybridization can induce artifacts, though comparison with non-denaturing datasets such as Hi-C and SPRITE indicates that the magnitude of the effects is small, as shown in Figure 1.

RESOURCE AVAILABILITY

Lead contact

Any request for more information or resources from this study should be directed to and will be fulfilled by the lead contact, Alistair N. Boettiger (boettiger@stanford.edu).

Materials availability

This study did not generate new unique reagents.

Data and code availability

- All processed ORCA data and unprocessed western blot images generated in this study have been deposited on Zenodo (https://doi.org/10.5281/zenodo.21014658) and are publicly available as of the date of this publication.
- This paper analyzes existing, publicly available data. Accession numbers or DOIs are listed in the key resources table.
- All original analysis code has been deposited on Zenodo (https://doi.org/10.5281/zenodo.21048398) and is publicly available as of the date of publication.
- Any additional information required to reanalyze the data reported in this paper is available from the lead contact upon request.

ACKNOWLEDGMENTS

We thank Ana Pombo (MDC, Berlin), Lonnie Welch (Ohio University), Tony Oro (Stanford University), and Ashby Morrison (Stanford University) for insightful discussion and feedback. We thank members of the Boettiger and Wang labs for feedback and critical reading of the text. We thank the Scientific Computing Core (Flatiron Institute) for providing generous computing power. This work was supported by grants from the NIH (U01 DK127419 and R01

GM157292), the NSF (EF2022182), and fellowships from the Arnold and Mabel Beckman Foundation and David and Lucile Packard Foundation to A.N.B.; the Walter and Idun Berry Foundation postdoctoral grant to A.H.; the NSF Graduate Research Fellowship Program (NSF DGE-1656518) to D.J.L.; and the Stanford SGF to A.R.V.

AUTHOR CONTRIBUTIONS

D.J.L., A.H., K.C.W., and A.N.B. designed the experiments. D.J.L. and A.H. performed the experiments. A.N. performed the western blot. D.J.L., A.H., A.R.V., S.G., and A.N.B. performed the analysis and interpreted the results, with support from T.U. D.J.L., A.H., A.R.V., K.C.W., and A.N.B. wrote the manuscript.

DECLARATION OF INTERESTS

The authors declare no competing interests.

DECLARATION OF GENERATIVE AI AND AI-ASSISTED TECHNOLOGIES IN THE WRITING PROCESS

During the preparation of this work, the authors used Claude and Gemini to assist in writing and debugging analysis code. After using these tools, the authors reviewed and edited the content and also take full responsibility for the content of the publication.

STAR★METHODS

Detailed methods are provided in the online version of this paper and include the following:

- KEY RESOURCES TABLE
- EXPERIMENTAL MODEL AND STUDY PARTICIPANT DETAILS
  - Cell lines and cell culture
- METHOD DETAILS
  - Perturbation treatments and sample preparation
  - Western blotting
  - Selecting SEs for oligopaint probe design
  - Linking SEs to genes
  - Oligopaints probe synthesis
  - Multiplex cell labeling and sample preparation
  - Primary probe hybridization
  - ORCA imaging
- QUANTIFICATION AND STATISTICAL ANALYSIS
  - Image analysis to identify SE coordinates in 3D
  - Perturbation ORCA image analysis
  - RNA quantification
  - *Sox2* SCR distance analysis
  - ORCA data analyses
  - ChIP-seq analysis
  - Processing Hi-C, SPRITE and GAM for ORCA validation
  - DamID analysis
  - RNA & DNA SPRITE analysis
  - Genomic distance analysis
  - Identifying SEs in cerebellum
  - seqFISH+ data analyses
  - Statistical analysis

SUPPLEMENTAL INFORMATION

Supplemental information can be found online at https://doi.org/10.1016/j.xgen.2026.101331.

Received: April 27, 2024
Revised: February 27, 2026
Accepted: July 20, 2026
Published: August 17, 2026

REFERENCES

1. Furlong, E.E.M., and Levine, M. (2018). Developmental enhancers and chromosome topology. Science *361*, 1341–1345. https://doi.org/10.1126/science.aau0320.
2. Inoue, F., and Ahituv, N. (2015). Decoding enhancers using massively parallel reporter assays. Genomics *106*, 159–164. https://doi.org/10.1016/j.ygeno.2015.06.005.
3. Catarino, R.R., and Stark, A. (2018). Assessing sufficiency and necessity of enhancer activities for gene expression and the mechanisms of transcription activation. Genes Dev. *32*, 202–223. https://doi.org/10.1101/gad.310367.117.
4. Mir, M., Bickmore, W., Furlong, E.E.M., and Narlikar, G. (2019). Chromatin topology, condensates and gene regulation: shifting paradigms or just a phase? Development *146*, dev182766. https://doi.org/10.1242/dev.182766.
5. Lim, B., and Levine, M.S. (2021). Enhancer-promoter communication: hubs or loops? Curr. Opin. Genet. Dev. *67*, 5–9. https://doi.org/10.1016/j.gde.2020.10.001.
6. Galouzis, C.C., and Furlong, E.E.M. (2022). Regulating specificity in enhancer-promoter communication. Curr. Opin. Cell Biol. *75*, 102065. https://doi.org/10.1016/j.ceb.2022.01.010.
7. Hamamoto, K., and Fukaya, T. (2022). Molecular architecture of enhancer-promoter interaction. Curr. Opin. Cell Biol. *74*, 62–70. https://doi.org/10.1016/j.ceb.2022.01.003.
8. Nollmann, M., Bennabi, I., Götz, M., and Gregor, T. (2022). The Impact of Space and Time on the Functional Output of the Genome. Cold Spring Harb. Perspect. Biol. *14*, a040378. https://doi.org/10.1101/cshperspect.a040378.
9. Zhao, J., and Faryabi, R.B. (2023). Spatial promoter-enhancer hubs in cancer: organization, regulation, and function. Trends Cancer *9*, 1069–1084. https://doi.org/10.1016/j.trecan.2023.07.017.
10. Oudelaar, A.M., Davies, J.O.J., Hanssen, L.L.P., Telenius, J.M., Schwessinger, R., Liu, Y., Brown, J.M., Downes, D.J., Chiariello, A.M., Bianco, S., et al. (2018). Single-allele chromatin interactions identify regulatory hubs in dynamic compartmentalized domains. Nat. Genet. *50*, 1744–1751. https://doi.org/10.1038/s41588-018-0253-2.
11. Ay, F., Vu, T.H., Zeitz, M.J., Varoquaux, N., Carette, J.E., Vert, J.-P., Hoffman, A.R., and Noble, W.S. (2015). Identifying multi-locus chromatin contacts in human cells using tethered multiple 3C. BMC Genom. *16*, 121. https://doi.org/10.1186/s12864-015-1236-7.
12. Darrow, E.M., Huntley, M.H., Dudchenko, O., Stamenova, E.K., Durand, N.C., Sun, Z., Huang, S.-C., Sanborn, A.L., Machol, I., Shamim, M., et al. (2016). Deletion of DXZ4 on the human inactive X chromosome alters higher-order genome architecture. Proc. Natl. Acad. Sci. USA *113*, E4504–E4512. https://doi.org/10.1073/pnas.1609643113.
13. Olivares-Chauvet, P., Mukamel, Z., Lifshitz, A., Schwartzman, O., Elkayam, N.O., Lubling, Y., Deikus, G., Sebra, R.P., and Tanay, A. (2016). Capturing pairwise and multi-way chromosomal conformations using chromosomal walks. Nature *540*, 296–300. https://doi.org/10.1038/nature20158.
14. Allahyar, A., Vermeulen, C., Bouwman, B.A.M., Krijger, P.H.L., Verstegen, M.J.A.M., Geeven, G., van Kranenburg, M., Pieterse, M., Straver, R., Haarhuis, J.H.I., et al. (2018). Enhancer hubs and loop collisions identified from single-allele topologies. Nat. Genet. *50*, 1151–1160. https://doi.org/10.1038/s41588-018-0161-5.
15. Deshpande, A.S., Ulahannan, N., Pendleton, M., Dai, X., Ly, L., Behr, J.M., Schwenk, S., Liao, W., Augello, M.A., Tyer, C., et al. (2022). Identifying synergistic high-order 3D chromatin conformations from genome-scale nanopore concatemer sequencing. Nat. Biotechnol. *40*, 1488–1499. https://doi.org/10.1038/s41587-022-01289-z.
16. Rao, S.S.P., Huang, S.-C., Glenn St Hilaire, B., Engreitz, J.M., Perez, E.M., Kieffer-Kwon, K.-R., Sanborn, A.L., Johnstone, S.E., Bascom, G.D.,

Bochkov, I.D., et al. (2017). Cohesin loss eliminates all loop domains. Cell *171*, 305–320.e24. https://doi.org/10.1016/j.cell.2017.09.026.

17. Beagrie, R.A., Scialdone, A., Schueler, M., Kraemer, D.C.A., Chotalia, M., Xie, S.Q., Barbieri, M., de Santiago, I., Lavitas, L.-M., Branco, M.R., et al. (2017). Complex multi-enhancer contacts captured by genome architecture mapping. Nature *543*, 519–524. https://doi.org/10.1038/nature21411.
18. Quinodoz, S.A., Ollikainen, N., Tabak, B., Palla, A., Schmidt, J.M., Detmar, E., Lai, M.M., Shishkin, A.A., Bhat, P., Takei, Y., et al. (2018). Higher-order inter-chromosomal hubs shape 3D genome organization in the nucleus. Cell *174*, 744–757.e24. https://doi.org/10.1016/j.cell.2018.05.024.
19. Whyte, W.A., Orlando, D.A., Hnisz, D., Abraham, B.J., Lin, C.Y., Kagey, M.H., Rahl, P.B., Lee, T.I., and Young, R.A. (2013). Master transcription factors and mediator establish super-enhancers at key cell identity genes. Cell *153*, 307–319. https://doi.org/10.1016/j.cell.2013.03.035.
20. Hnisz, D., Abraham, B.J., Lee, T.I., Lau, A., Saint-André, V., Sigova, A.A., Hoke, H.A., and Young, R.A. (2013). Super-enhancers in the control of cell identity and disease. Cell *155*, 934–947. https://doi.org/10.1016/j.cell.2013.09.053.
21. Sabari, B.R., Dall'Agnese, A., Boija, A., Klein, I.A., Coffey, E.L., Shrinivas, K., Abraham, B.J., Hannett, N.M., Zamudio, A.V., Manteiga, J.C., et al. (2018). Coactivator condensation at super-enhancers links phase separation and gene control. Science *361*, eaar3958. https://doi.org/10.1126/science.aar3958.
22. Imakaev, M., Fudenberg, G., McCord, R.P., Naumova, N., Goloborodko, A., Lajoie, B.R., Dekker, J., and Mirny, L.A. (2012). Iterative correction of Hi-C data reveals hallmarks of chromosome organization. Nat. Methods *9*, 999–1003. https://doi.org/10.1038/nmeth.2148.
23. Akgol Oksuz, B., Yang, L., Abraham, S., Venev, S.V., Krietenstein, N., Parsi, K.M., Ozadam, H., Oomen, M.E., Nand, A., Mao, H., et al. (2021). Systematic evaluation of chromosome conformation capture assays. Nat. Methods *18*, 1046–1055. https://doi.org/10.1038/s41592-021-01248-7.
24. Uyehara, C.M., and Apostolou, E. (2023). 3D enhancer-promoter interactions and multi-connected hubs: Organizational principles and functional roles. Cell Rep. *42*, 112068. https://doi.org/10.1016/j.celrep.2023.112068.
25. Arrastia, M.V., Jachowicz, J.W., Ollikainen, N., Curtis, M.S., Lai, C., Quinodoz, S.A., Selck, D.A., Ismagilov, R.F., and Guttman, M. (2022). Single-cell measurement of higher-order 3D genome organization with scSPRITE. Nat. Biotechnol. *40*, 64–73. https://doi.org/10.1038/s41587-021-00998-1.
26. Tan, L., Shi, J., Moghadami, S., Parasar, B., Wright, C.P., Seo, Y., Vallejo, K., Cobos, I., Duncan, L., Chen, R., and Deisseroth, K. (2023). Lifelong restructuring of 3D genome architecture in cerebellar granule cells. Science *381*, 1112–1119. https://doi.org/10.1126/science.adh3253.
27. Tan, L., Ma, W., Wu, H., Zheng, Y., Xing, D., Chen, R., Li, X., Daley, N., Deisseroth, K., and Xie, X.S. (2021). Changes in genome architecture and transcriptional dynamics progress independently of sensory experience during post-natal brain development. Cell *184*, 741–758.e17. https://doi.org/10.1016/j.cell.2020.12.032.
28. Lando, D., Ma, X., Cao, Y., Jartseva, A., Stevens, T.J., Boucher, W., Reynolds, N., Montibus, B., Hall, D., Lackner, A., et al. (2024). Enhancer-promoter interactions are reconfigured through the formation of long-range multiway hubs as mouse ES cells exit pluripotency. Mol. Cell *84*, 1406–1421.e8. https://doi.org/10.1016/j.molcel.2024.02.015.
29. Liu, Z., Chen, Y., Xia, Q., Liu, M., Xu, H., Chi, Y., Deng, Y., and Xing, D. (2023). Linking genome structures to functions by simultaneous single-cell Hi-C and RNA-seq. Science *380*, 1070–1076. https://doi.org/10.1126/science.adg3797.
30. Zhou, T., Zhang, R., Jia, D., Doty, R.T., Munday, A.D., Gao, D., Xin, L., Abkowitz, J.L., Duan, Z., and Ma, J. (2024). GAGE-seq concurrently profiles multiscale 3D genome organization and gene expression in single cells. Nat. Genet. *56*, 1701–1711. https://doi.org/10.1038/s41588-024-01745-3.
31. Quinodoz, S.A., Jachowicz, J.W., Bhat, P., Ollikainen, N., Banerjee, A.K., Goronzy, I.N., Blanco, M.R., Chovanec, P., Chow, A., Markaki, Y., et al. (2021). RNA promotes the formation of spatial compartments in the nucleus. Cell *184*, 5775–5790.e30. https://doi.org/10.1016/j.cell.2021.10.014.
32. Wen, X., Luo, Z., Zhao, W., Calandrelli, R., Nguyen, T.C., Wan, X., Charles Richard, J.L., and Zhong, S. (2024). Single-cell multiplex chromatin and RNA interactions in ageing human brain. Nature *628*, 648–656. https://doi.org/10.1038/s41586-024-07239-w.
33. Takei, Y., Yun, J., Zheng, S., Ollikainen, N., Pierson, N., White, J., Shah, S., Thomassie, J., Suo, S., Eng, C.-H.L., et al. (2021). Integrated spatial genomics reveals global architecture of single nuclei. Nature *590*, 344–350. https://doi.org/10.1038/s41586-020-03126-2.
34. Takei, Y., Yang, Y., White, J., Goronzy, I.N., Yun, J., Prasad, M., Ombelets, L.J., Schindler, S., Bhat, P., Guttman, M., and Cai, L. (2025). Spatial multi-omics reveals cell-type-specific nuclear compartments. Nature *641*, 1037–1047. https://doi.org/10.1038/s41586-025-08838-x.
35. Bhat, P., Chow, A., Emert, B., Ettlin, O., Quinodoz, S.A., Strehle, M., Takei, Y., Burr, A., Goronzy, I.N., Chen, A.W., et al. (2024). Genome organization around nuclear speckles drives mRNA splicing efficiency. Nature *629*, 1165–1173. https://doi.org/10.1038/s41586-024-07429-6.
36. Su, J.-H., Zheng, P., Kinrot, S.S., Bintu, B., and Zhuang, X. (2020). Genome-Scale Imaging of the 3D Organization and Transcriptional Activity of Chromatin. Cell *182*, 1641–1659.e26. https://doi.org/10.1016/j.cell.2020.07.032.
37. Kawasaki, K., and Fukaya, T. (2023). Functional coordination between transcription factor clustering and gene activity. Mol. Cell *83*, 1605–1622.e9. https://doi.org/10.1016/j.molcel.2023.04.018.
38. Du, M., Stitzinger, S.H., Spille, J.-H., Cho, W.-K., Lee, C., Hijaz, M., Quintana, A., and Cissé, I.I. (2024). Direct observation of a condensate effect on super-enhancer controlled gene bursting. Cell *187*, 331–344.e17. https://doi.org/10.1016/j.cell.2023.12.005.
39. Mateo, L.J., Murphy, S.E., Hafner, A., Cinquini, I.S., Walker, C.A., and Boettiger, A.N. (2019). Visualizing DNA folding and RNA in embryos at single-cell resolution. Nature *568*, 49–54. https://doi.org/10.1038/s41586-019-1035-4.
40. Sethi, A., Gu, M., Gumusgoz, E., Chan, L., Yan, K.-K., Rozowsky, J., Barozzi, I., Afzal, V., Akiyama, J.A., Plajzer-Frick, I., et al. (2020). Supervised enhancer prediction with epigenetic pattern recognition and targeted validation. Nat. Methods *17*, 807–814. https://doi.org/10.1038/s41592-020-0907-8.
41. Bonev, B., Mendelson Cohen, N., Szabo, Q., Fritsch, L., Papadopoulos, G.L., Lubling, Y., Xu, X., Lv, X., Hugnot, J.-P., Tanay, A., and Cavalli, G. (2017). Multiscale 3D Genome Rewiring during Mouse Neural Development. Cell *171*, 557–572.e24. https://doi.org/10.1016/j.cell.2017.09.043.
42. Huang, H., Zhu, Q., Jussila, A., Han, Y., Bintu, B., Kern, C., Conte, M., Zhang, Y., Bianco, S., Chiariello, A.M., et al. (2021). CTCF mediates dosage- and sequence-context-dependent transcriptional insulation by forming local chromatin domains. Nat. Genet. *53*, 1064–1074. https://doi.org/10.1038/s41588-021-00863-6.
43. Zhou, H.Y., Katsman, Y., Dhaliwal, N.K., Davidson, S., Macpherson, N.N., Sakthidevi, M., Collura, F., and Mitchell, J.A. (2014). A Sox2 distal enhancer cluster regulates embryonic stem cell differentiation potential. Genes Dev. *28*, 2699–2711. https://doi.org/10.1101/gad.248526.114.
44. Li, Y., Rivera, C.M., Ishii, H., Jin, F., Selvaraj, S., Lee, A.Y., Dixon, J.R., and Ren, B. (2014). CRISPR reveals a distal super-enhancer required for Sox2 expression in mouse embryonic stem cells. PLoS One *9*, e114485. https://doi.org/10.1371/journal.pone.0114485.
45. Blinka, S., Reimer, M.H., Jr., Pulakanti, K., and Rao, S. (2016). Super-Enhancers at the Nanog Locus Differentially Regulate Neighboring Pluripotency-Associated Genes. Cell Rep. *17*, 19–28. https://doi.org/10.1016/j.celrep.2016.09.002.

46. Shen, Y., Yue, F., McCleary, D.F., Ye, Z., Edsall, L., Kuan, S., Wagner, U., Dixon, J., Lee, L., Lobanenkov, V.V., and Ren, B. (2012). A map of the cis-regulatory sequences in the mouse genome. Nature *488*, 116–120. https://doi.org/10.1038/nature11243.

47. Hnisz, D., Shrinivas, K., Young, R.A., Chakraborty, A.K., and Sharp, P.A. (2017). A phase separation model for transcriptional control. Cell *169*, 13–23. https://doi.org/10.1016/j.cell.2017.02.007.

48. Pei, G., Lyons, H., Li, P., and Sabari, B.R. (2025). Transcription regulation by biomolecular condensates. Nat. Rev. Mol. Cell Biol. *26*, 213–236. https://doi.org/10.1038/s41580-024-00789-x.

49. Hafner, A., Park, M., Berger, S.E., Murphy, S.E., Nora, E.P., and Boettiger, A.N. (2023). Loop stacking organizes genome folding from TADs to chromosomes. Mol. Cell *83*, 1377–1392.e6. https://doi.org/10.1016/j.molcel.2023.04.008.

50. Cho, W.-K., Spille, J.-H., Hecht, M., Lee, C., Li, C., Grube, V., and Cisse, I.I. (2018). Mediator and RNA polymerase II clusters associate in transcription-dependent condensates. Science *361*, 412–415. https://doi.org/10.1126/science.aar4199.

51. Harper, N.W., Birdsall, G.A., Honeywell, M.E., Ward, K.M., Pai, A.A., and Lee, M.J. (2025). RNA Pol II inhibition activates cell death independently from the loss of transcription. Cell *188*, 6301–6316.e29. https://doi.org/10.1016/j.cell.2025.07.034.

52. Di Giammartino, D.C., Polyzos, A., and Apostolou, E. (2020). Transcription factors: building hubs in the 3D space. Cell Cycle *19*, 2395–2410. https://doi.org/10.1080/15384101.2020.1805238.

53. Boija, A., Klein, I.A., Sabari, B.R., Dall'Agnese, A., Coffey, E.L., Zamudio, A.V., Li, C.H., Shrinivas, K., Manteiga, J.C., Hannett, N.M., et al. (2018). Transcription Factors Activate Genes through the Phase-Separation Capacity of Their Activation Domains. Cell *175*, 1842–1855.e16. https://doi.org/10.1016/j.cell.2018.10.042.

54. Shrinivas, K., Sabari, B.R., Coffey, E.L., Klein, I.A., Boija, A., Zamudio, A.V., Schuijers, J., Hannett, N.M., Sharp, P.A., Young, R.A., and Chakraborty, A.K. (2019). Enhancer Features that Drive Formation of Transcriptional Condensates. Mol. Cell *75*, 549–561.e7. https://doi.org/10.1016/j.molcel.2019.07.009.

55. Dong, P., Zhang, S., Gandin, V., Xie, L., Wang, L., Lemire, A.L., Li, W., Otsuna, H., Kawase, T., Lander, A.D., et al. (2024). Cohesin prevents cross-domain gene coactivation. Nat. Genet. *56*, 1654–1664. https://doi.org/10.1038/s41588-024-01852-1.

56. Zhang, S., Übelmesser, N., Josipovic, N., Forte, G., Slotman, J.A., Chiang, M., Gothe, H.J., Gusmao, E.G., Becker, C., Altmüller, J., et al. (2021). RNA polymerase II is required for spatial chromatin reorganization following exit from mitosis. Sci. Adv. *7*, eabg8205. https://doi.org/10.1126/sciadv.abg8205.

57. Zhang, S., Übelmesser, N., Barbieri, M., and Papantonis, A. (2023). Enhancer-promoter contact formation requires RNAPII and antagonizes loop extrusion. Nat. Genet. *55*, 832–840. https://doi.org/10.1038/s41588-023-01364-4.

58. Karpinska, M.A., Zhu, Y., Fakhraei Ghazvini, Z., Ramasamy, S., Barbieri, M., Cao, T.B.N., Varahram, N., Aljahani, A., Lidschreiber, M., Papantonis, A., and Oudelaar, A.M. (2025). CTCF depletion decouples enhancer-mediated gene activation from chromatin hub formation. Nat. Struct. Mol. Biol. *32*, 1268–1281. https://doi.org/10.1038/s41594-025-01555-z.

59. Khanna, N., Zhang, Y., Lucas, J.S., Dudko, O.K., and Murre, C. (2019). Chromosome dynamics near the sol-gel phase transition dictate the timing of remote genomic interactions. Nat. Commun. *10*, 2771.

60. Alekseyenko, A.A., Walsh, E.M., Wang, X., Grayson, A.R., Hsi, P.T., Kharchenko, P.V., Kuroda, M.I., and French, C.A. (2015). The oncogenic BRD4-NUT chromatin regulator drives aberrant transcription within large topological domains. Genes Dev. *29*, 1507–1523. https://doi.org/10.1101/gad.267583.115.

61. Rosencrance, C.D., Ammouri, H.N., Yu, Q., Ge, T., Rendleman, E.J., Marshall, S.A., and Eagen, K.P. (2020). Chromatin Hyperacetylation Impacts Chromosome Folding by Forming a Nuclear Subcompartment. Mol. Cell *78*, 112–126.e12. https://doi.org/10.1016/j.molcel.2020.03.018.

62. Ahn, J.H., Davis, E.S., Daugird, T.A., Zhao, S., Quiroga, I.Y., Uryu, H., Li, J., Storey, A.J., Tsai, Y.-H., Keeley, D.P., et al. (2021). Phase separation drives aberrant chromatin looping and cancer development. Nature *595*, 591–595. https://doi.org/10.1038/s41586-021-03662-5.

63. Quiroga, I.Y., Ahn, J.H., Wang, G.G., and Phanstiel, D. (2022). Oncogenic fusion proteins and their role in three-dimensional chromatin structure, phase separation, and cancer. Curr. Opin. Genet. Dev. *74*, 101901. https://doi.org/10.1016/j.gde.2022.101901.

64. Sanalkumar, R., Dong, R., Lee, L., Xing, Y.-H., Iyer, S., Letovanec, I., La Rosa, S., Finzi, G., Musolino, E., Papait, R., et al. (2023). Highly connected 3D chromatin networks established by an oncogenic fusion protein shape tumor cell identity. Sci. Adv. *9*, eabo3789. https://doi.org/10.1126/sciadv.abo3789.

65. Petrovic, J., Zhou, Y., Fasolino, M., Goldman, N., Schwartz, G.W., Mumbach, M.R., Nguyen, S.C., Rome, K.S., Sela, Y., Zapataro, Z., et al. (2019). Oncogenic Notch Promotes Long-Range Regulatory Interactions within Hyperconnected 3D Cliques. Mol. Cell *73*, 1174–1190.e12. https://doi.org/10.1016/j.molcel.2019.01.006.

66. Zhao, J., Zhou, Y., Tzelepis, I., Burget, N.G., Shi, J., and Faryabi, R.B. (2024). Oncogenic transcription factors instruct promoter-enhancer hubs in individual triple negative breast cancer cells. Sci. Adv. *10*, eadl4043. https://doi.org/10.1126/sciadv.adl4043.

67. Breves, S.L., Di Giammartino, D.C., Nicholson, J., Cirigliano, S., Mahmood, S.R., Lee, U.J., Martinez-Fundichely, A., Jungverdorben, J., Singhania, R., Rajkumar, S., et al. (2025). Three-dimensional regulatory hubs support oncogenic programs in glioblastoma. Mol. Cell *85*, 1330–1348.e6. https://doi.org/10.1016/j.molcel.2025.03.007.

68. Zhou, Y., Petrovic, J., Zhao, J., Zhang, W., Bigdeli, A., Zhang, Z., Berger, S.L., Pear, W.S., and Faryabi, R.B. (2022). EBF1 nuclear repositioning instructs chromatin refolding to promote therapy resistance in T leukemic cells. Mol. Cell *82*, 1003–1020.e15.

69. Kim, S., and Wysocka, J. (2023). Deciphering the multi-scale, quantitative cis-regulatory code. Mol. Cell *83*, 373–392. https://doi.org/10.1016/j.molcel.2022.12.032.

70. Chen, L.-F., Lee, J., and Boettiger, A. (2023). Recent progress and challenges in single-cell imaging of enhancer-promoter interaction. Curr. Opin. Genet. Dev. *79*, 102023.

71. Sun, F., Chronis, C., Kronenberg, M., Chen, X.-F., Su, T., Lay, F.D., Plath, K., Kurdistani, S.K., and Carey, M.F. (2019). Promoter-Enhancer Communication Occurs Primarily within Insulated Neighborhoods. Mol. Cell *73*, 250–263.e5. https://doi.org/10.1016/j.molcel.2018.10.039.

72. Liu, N.Q., Magnitov, M., Schijns, M.M.G.A., van Schaik, T., Teunissen, H., van Steensel, B., and de Wit, E. (2025). Extrusion fountains are restricted by WAPL-dependent cohesin release and CTCF barriers. Nucleic Acids Res. *53*, gkaf549. https://doi.org/10.1093/nar/gkaf549.

73. Stringer, C., Wang, T., Michaelos, M., and Pachitariu, M. (2021). Cellpose: a generalist algorithm for cellular segmentation. Nat. Methods *18*, 100–106. https://doi.org/10.1038/s41592-020-01018-x.

74. Quinlan, A.R., and Hall, I.M. (2010). BEDTools: a flexible suite of utilities for comparing genomic features. Bioinformatics *26*, 841–842. https://doi.org/10.1093/bioinformatics/btq033.

75. Langmead, B., and Salzberg, S.L. (2012). Fast gapped-read alignment with Bowtie 2. Nat. Methods *9*, 357–359. https://doi.org/10.1038/nmeth.1923.

76. Zhao, H., Sun, Z., Wang, J., Huang, H., Kocher, J.-P., and Wang, L. (2014). CrossMap: a versatile tool for coordinate conversion between genome assemblies. Bioinformatics *30*, 1006–1007. https://doi.org/10.1093/bioinformatics/btt730.

77. Ramírez, F., Ryan, D.P., Grüning, B., Bhardwaj, V., Kilpert, F., Richter, A.S., Heyne, S., Dündar, F., and Manke, T. (2016). deepTools2: a next

CellPress
OPEN ACCESS

generation web server for deep-sequencing data analysis. Nucleic Acids Res. *44*, W160–W165. https://doi.org/10.1093/nar/gkw257.

78. Zhang, Y., Liu, T., Meyer, C.A., Eeckhoute, J., Johnson, D.S., Bernstein, B.E., Nusbaum, C., Myers, R.M., Brown, M., Li, W., and Liu, X.S. (2008). Model-based analysis of ChIP-Seq (MACS). Genome Biol. *9*, R137. https://doi.org/10.1186/gb-2008-9-9-r137.

79. Mateo, L.J., Sinnott-Armstrong, N., and Boettiger, A.N. (2021). Tracing DNA paths and RNA profiles in cultured cells and tissues with ORCA. Nat. Protoc. *16*, 1647–1713. https://doi.org/10.1038/s41596-020-00478-x.

80. Kraft, K., Yost, K.E., Murphy, S.E., Magg, A., Long, Y., Corces, M.R., Granja, J.M., Wittler, L., Mundlos, S., Cech, T.R., et al. (2022). Polycomb-mediated genome architecture enables long-range spreading of H3K27 methylation. Proc. Natl. Acad. Sci. USA *119*, e2201883119. https://doi.org/10.1073/pnas.2201883119.

81. Rigano, A., Ehmsen, S., Öztürk, S.U., Ryan, J., Balashov, A., Hammer, M., Kirli, K., Boehm, U., Brown, C.M., Bellve, K., et al. (2021). Micro-Meta App: an interactive tool for collecting microscopy metadata based on community specifications. Nat. Methods *18*, 1489–1495. https://doi.org/10.1038/s41592-021-01315-z.

STAR★METHODS

KEY RESOURCES TABLE

REAGENT or RESOURCE	SOURCE	IDENTIFIER
**Antibodies**		
Rabbit polyclonal anti-Rad21 antibody	Abcam	Cat# ab154769,; RRID:AB_2783833
Rabbit monoclonal anti-CTCF antibody [EPR7314(B)]	Abcam	Cat# ab128873; RRID:AB_11144295
Rabbit monoclonal beta-Actin antibody (13E5)	Cell Signaling Technology	Cat# CST 5125S; RRID: AB_1903890
**Chemicals, peptides, and recombinant proteins**		
indole-3-acetic acid (IAA)	Sigma-Aldrich	Cat# I2886
(+)-JQ1	MedChemExpress	Cat# HY-13030
Triptolide	MedChemExpress	Cat# HY-32735
TCO-PEG4-TFP Ester	Click Chemistry Tools	Cat# CCT-1398-2
Methyltetrazine-PEG4-Amine	Click Chemistry Tools	Cat# CCT-1012-100
Methyltetrazine-NHS Ester	Click Chemistry Tools	Cat# CCT-1128-25
**Deposited data**		
Processed ORCA data	This paper	https://doi.org/10.5281/zenodo.21014658
Unprocessed western blot images	This paper	https://doi.org/10.5281/zenodo.21014658
Sox2 SCR FISH	Huang et al.[42]	4DN Data Portal: https://data.4dnucleome.org/publications/4f766397-5ead-416e-b891-1c95391dcd77/#expsets-table
Two-layer DNA seqFISH+	Takei et al.[34]	https://doi.org/10.5281/zenodo.7693825
Intron seqFISH+	Takei et al.[34]	https://doi.org/10.5281/zenodo.7693825
OCT4 ChIP-seq	Whyte et al.[19]	GEO: GSM1082340
SOX2 ChIP-seq	Whyte et al.[19]	GEO: GSM1082341
NANOG ChIP-seq	Whyte et al.[19]	GEO: GSM1082342
MED1 ChIP-seq	Sun et al.[71]	GEO: GSM3175679, GSM3175678
RNA Pol II ChIP-seq	Sun et al.[71]	GEO: GSM3175687, GSM3175686
H3K27ac ChIP-seq	ENCODE	GEO: GSM1000126
DamID	Liu et al.[72]	GEO: GSE181693
RNA & DNA SPRITE	Quinodoz et al.[31]	GEO: GSE151515
Hi-C	Bonev et al.[41]	GEO: GSE161259
SPRITE	Quinodoz et al.[18]	GEO: GSE114242
GAM	Beagrie et al.[17]	GEO: GSE64881
H3K27ac ChIP-seq (adult mouse cerebellum)	ENCODE	GEO: GSM1000097
**Experimental models: Cell lines**		
Mouse: ES-E14TG2a (E14)	ATCC	ATCC Cat# CRL-1821; RRID:CVCL_9108
ES-E14TG2a CTCF-AID (clone)	Gift from Elphege Nora	clone EN52.9.1
ES-E14TG2a RAD21-AID (clone)	Gift from Elphege Nora	clone EN272.2
**Oligonucleotides**		
ORCA oligopool	This paper	https://doi.org/10.5281/zenodo.21014658
Cell barcode oligos	This paper	https://doi.org/10.5281/zenodo.21014658
**Software and algorithms**		
ORCA spot calling analysis	This paper	https://doi.org/10.5281/zenodo.7698978 and https://doi.org/10.5281/zenodo.21048398
CellPose cell segmentation	Stringer et al.[73]	N/A
bedtools	Quinlan and Hall[74]	https://github.com/arq5x/bedtools2
bowtie2	Langmead and Salzberg[75]	https://github.com/BenLangmead/bowtie2
Trim Galore	Felix Krueger	https://github.com/FelixKrueger/TrimGalore

(*Continued on next page*)

Continued

REAGENT or RESOURCE	SOURCE	IDENTIFIER
CrossMap	Zhao et al.[76]	https://github.com/liguowang/CrossMap
deeptools	Ramírez et al.[77]	https://github.com/deeptools/deepTools
Macs2	Zhang et al.[78]	https://github.com/macs3-project/MACS

EXPERIMENTAL MODEL AND STUDY PARTICIPANT DETAILS

Cell lines and cell culture

Wild-type ES-E14TG2a (XY, 129/Ola) mouse embryonic stem cells (E14 mESCs) were obtained from ATCC (CRL-1821, CVCL_9108) and adapted to grow without the assistance of feeder cells. Degron E14 mESC lines RAD21-AID (clone EN272.2) and CTCF-AID (clone EN52.9.1) were gifted from Elphege Nora. All E14 cell lines were cultured in media containing 500 mL of KnockOut DMEM (Thermo Fisher 10829018), 75 mL ES-grade FBS (Thermo Fisher 16141079), 5 mL of MEM NEAA (Thermo Fisher 11140050), 5 mL HEPES (1M) (Thermo Fisher 15630080), 500 μL 2-Mercaptoethanol (55 mM) (Thermo Fisher 21985023), 5 mL GlutaMAX (Thermo Fisher 35050061), 5 mL of penicillin-streptomycin (Thermo Fisher 15140122), and 50 μL of mouse LIF (10ˆ7 U/mL) (MilliporeSigma ESG1107). 6-well plates or 10 cm dishes were coated with 0.1% gelatin (MilliporeSigma SF008) for at least 10 min at RT before use. Media was changed daily, and cells were passaged every 2 days at a dilution ratio of 1:5-1:10.

METHOD DETAILS

Perturbation treatments and sample preparation

Triptolide and (+)-JQ1 were obtained at 10 mM stocks in DMSO (MedChemExpress, HY-32735 and HY-13030). Triptolide and JQ1 working stocks were prepared at 1 mM in DMSO and then diluted 1:1,000 to a final concentration of 1 μM in media, with 0.1% DMSO as control. WT E14 mESCs were treated for 4 h for triptolide, 6 h for JQ1, and 6 h for DMSO before collection. For RAD21 and CTCF AID-mediated degradation, indole-3-acetic acid (IAA) was resuspended in DMSO at a stock concentration of 1M (Sigma i2886). IAA was then diluted 1:2,000 in media to a final concentration of 500 μM, with 0.05% DMSO as control. RAD21 AID-tagged or CTCF-AID-tagged E14 mESCs were treated with IAA or DMSO for 4 h before collection. For all treatments, drug or DMSO controls were diluted in warmed, fresh media and a complete media change was performed.

For all ORCA experiments, cells collected from live cell culture were detached from plates with trypsin/EDTA, fixed with 4% formaldehyde (Electron Microscopy Sciences 15714), and stored in 70% ethanol for up to 6 months as previously described.[49]

Western blotting

Cells were lysed on ice for 15 min in RIPA buffer supplemented with a 1× Protease inhibitor cocktail, with occasional vortexing. Lysates were cleared by centrifugation at 20,000 *g* for 10 min at 4°C. The cleared lysates were mixed with protein loading buffer and boiled at 95°C for 5 min. For each well, protein extracts derived from 250,000 cells were loaded onto 4–12% Novex™ Tris-Glycine Mini Protein precast gels, separated in Tris-Glycine-SDS running buffer, and transferred onto nitrocellulose membrane. The membrane was stained with Ponceau S to visualize total protein loading. After blocking for 1 h at room temperature in TBST buffer supplemented with 5% non-fat dry milk, the blots were probed overnight at 4°C with Rad21 (Abcam 154769, 1:1000) or CTCF (Abcam 128873, 1:1000) and Beta-Actin (CST 5125S, 1:3000) antibodies in blocking buffer. Membranes were then washed three times for 5 min each in TBST buffer at room temperature and incubated with the secondary antibody (Goat Anti-Rabbit-IgG-HRP, Biorad 1706515, diluted 1:3000 in blocking buffer) for 1 h at room temperature. After three washes of 15 min each in TBST, samples were imaged on the ChemiDoc XRS+ imager with Clarity Western ECL Blotting Substrates (Bio-Rad).

Selecting SEs for oligopaint probe design

A total of 376 target SEs were chosen by including all 231 mESC-related SEs from Whyte et al. (2013)[19] and an additional 145 SEs, sorted by chromosome from Sabari et al. (2018).[21] Two probes were duplicates, resulting in a total of 374 unique target SEs. For probe design, a standardized target region of 15 kb was chosen for every SE, calculated by choosing a region 7.5 kb 5′ and 7.5 kb 3′ of the center of the published SE coordinate region (mm9). For SEs with overlapping regions in both publications, the coordinates from Whyte et al. (2013)[19] were used. Since the SEs for *Hist1h2bg* and *Hist1h2ad* were within 15 kb of each other, the SE regions were merged and re-labelled as *Hist1h2bf*. The SEs for *Snord2* and *Eif4a2* were also merged due to linear proximity and labeled under *Eif4a2*. The resulting coordinates were then converted to mm10 using the UCSC Liftover tool, to facilitate comparisons with other datasets mapped to mm10.

Linking SEs to genes

Gene assignments for each SE were derived from their original publication, Whyte et al. (2013)[19] or Sabari et al. (2018).[21] Briefly, both studies linked genes based on the nearest, expressed RefSeq transcript to the center of each SE.

Oligopaints probe synthesis

The probe synthesis was performed as described before.[39,79,80] Briefly, oligopaints were amplified by limited-cycle PCR. *In vitro* transcription with T7 RNA polymerase was performed to generate ssRNA. Reverse transcription with Maxima H Minus RT enzyme was then performed to convert the ssRNA to ssDNA probes.

Multiplex cell labeling and sample preparation

Fixed cells that were stored in 70% ethanol/PBS solution were barcoded with sample-specific oligos as previously described.[49] Briefly, 3′ amine-modified oligonucleotides were conjugated to methyltetrazine. TCO-PEG4-TFP Ester was then used to label the cell surface for each condition with a specific methyltetrazine oligonucleotide. After the labeling procedure, barcoded cells from multiple conditions were pooled together for simultaneous imaging. Pooled cells were plated on poly-(D)-lysine coated glass coverslips. After 3× washes with 1× PBS, cells were permeabilized for 10 min with 0.5% Triton X-100 solution in 1× PBS followed by 2× washes with 1× PBS.

Primary probe hybridization

For DNA imaging preparation only, cells were incubated for exactly 5 min in 0.1M HCl solution, washed 3× with 1× PBS, and then incubated with RNAse A solution for 30 min at 37°C. After prior treatments, cells were then washed 3× in 2× SSC and then incubated for at least 1 h at RT in a solution of 2× SSC, 50% formamide, 10% dextran sulfate, and 0.1% Tween 20. This solution was completely aspirated from the coverslip and then replaced with 50 μg of DNA primary probe (10 μg/μL) that was mixed with 30 μL of 2× SSC, 50% formamide, 10% dextran sulfate, and 0.1% Tween 20. A small, 18 mm × 18 mm Sigmacote-treated coverslip was placed on top of the probes and cells, and the entire sample was incubated on a 90°C heat block surface for 3 min. Afterward, the sample was placed in a hybridization chamber and incubated at 42°C overnight. After incubation, the sample was incubated in 42°C 2× SSC for 10 min before the 18 mm × 18 mm coverslip was removed. This was followed by another 10 min incubation in 2× SSC at 42°C. The samples were washed 1× more in RT 2× SSC and then incubated in a fixation buffer (8% PFA, 2% glutaraldehyde, in 1× PBS) for 30 min at RT. A final 3× wash with 2× SSC was performed before the sample was ready for imaging.

For RNA imaging, cells were prepared similarly to DNA imaging with the exclusion of HCl and RNAse A incubation steps, 90°C incubation steps, and fixation after overnight 42°C incubation. 4 μg of RNA primary probe was used for incubation. After RNA imaging, cells were washed 3× in 1× PBS, re-permeabilized for 10 min with 0.5% Triton X-100 solution in 1× PBS, and followed by 2× washes with 1× PBS. The DNA imaging protocol was then followed.

ORCA imaging

Samples were imaged using a custom-built microscopy and microfluidics setup that was previously described.[79] The microscope configuration is documented in Micro-Meta App[81] and publicly available through the 4DN data portal: https://data.4dnucleome.org/microscope-configurations/84d52890-7e46-4cf8-810c-071ed0673ce8/.

For cell registration and drift correction between rounds of imaging, we used a fiducial signal. Similar to previous ORCA studies, we designed the fiducial to bind to all primary probes in the library, which was hybridized against the 5′ end of the primary probes to increase the overall nuclear punctate signal in the 561 nm laser channel. Secondary oligos conjugated to dyes that fluoresce in 647 nm (Cy5) and 750 nm (Alexa Fluor 750) were used for tag-specific imaging (IDT). Chromatic aberrations between the 647 and 750 channels were measured by performing replicate measurements with inverted labels for 4 of the SE pairs to accurately capture the aberrations at the appropriate focal plane and context of the cells.

QUANTIFICATION AND STATISTICAL ANALYSIS

Image analysis to identify SE coordinates in 3D

A new image analysis pipeline was adapted from our earlier ORCA approach[79] to accommodate the genome-wide nature of our probeset and the resulting challenges in disambiguating alleles. Rather than use a fixed distance from the center of a target locus as a filter for identifying spots and segregating alleles, the revised approach used the entire nucleus as the search area. The major steps are described below. These were implemented in MATLAB(TM) R2022a, and the functions and recipes for the analysis are included in the Github repository associated with our manuscript: https://github.com/BoettigerLab/SE-clustering-2026 (https://doi.org/10.5281/zenodo.21048398).

Drift correction

First, the 647 channel and 750 channel images from each round of hybridization were drift-corrected by aligning a fiducial probe that labeled the entire nucleus using a function that finds the displacement which maximizes the auto-correlation of each image with the first one taken. Then we used the same fiducial marker to segment the individual cells using the Cellpose algorithm.[73] Fine scale drift correction was then computed on a cell-by-cell basis, to allow for cell motion independent of the global coverslip drift.

Spot finding

For each drift-corrected image of each cell in each field of view and each chromatic channel, we selected the brightest two foci that matched the point-spread function (PSF) of our microscope and fit the centroid of these spots to a 3D Gaussian function using a least-squares approach.

Chromatic correction

After all the aligned spots were identified, we used our chromatic correction replicate images to specify all pairs of spots that were imaged first in 647 and then in 750 in different labeling rounds. Points which had no matched partner within 1 μm (due to variation in detection or background signal) were rejected, so that all retained points had a unique matched pair in the other channel. We then used these pairs to compute a 3D 2nd-order polynomial transformation that minimized the distance between all pairs. This function provides a measure of the chromatic aberrations in our microscope remaining after the built-in chromatic corrections provided by our Plan Apo correcting lens. This is shown as a vector field in Figure S1, which also shows the measured 3D displacement before and after correction, which improves from a median of 131 nm down to 63 nm (See Figures S1A–S1C).

Quantification of residual measurement error and labeling efficiency

We compared the measured 3D positions of SEs recorded in the first few rounds of labeling and imaging to their 3D positions measured several weeks later, at the end of ∼200 rounds of imaging, to quantify the accumulated uncertainty due to accumulated sample degradation, camera noise, labeling background changes, and uncorrected residual drift. This was a little less than 100 nm (precise values and the distribution of errors are shown in Figure S1D). For labeling efficiency, we computed for each SE the frequency with which both alleles were detected in every cell, and found most SEs to be detected at >90% of the time (Figure S1E). We also computed the number of detected SE alleles per cell (Figure S1F).

Allele assignments

After all points have been identified in 3D to sub-diffraction position, we assigned alleles to two distinct territories per chromosome by minimizing the intra-chromosomal distances between all alleles assigned to the same chromosome. All SEs assigned to the same homolog are *cis*-linked, and any interaction of these SEs with any other SE are by definition *trans*-contacts. This method does not specifically distinguish maternal vs. paternal alleles but does prevent inter-SE distances being inflated by accidentally averaging in *trans*-distances as well. The resulting 3D localizations with SE assignments and allele assignments are recorded in the core FOF-CT table (see Data Availability).

Perturbation ORCA image analysis

SE selection

A total of 112 SEs focused on chr 2, 3, 6, and 11 across control and perturbed cells were imaged with both Cy5-and Alexa Fluor 750-conjugated secondary oligos. Due to technical issues, we excluded Cy5-labelled SEs in the perturbation image analysis but still chromatically corrected 750 nm and 647 nm data, resulting in 80 SEs (Table S3). Of the 80 remaining SEs, 6 SEs that appeared to have cross-reactive effects in both the wild-type and perturbation imaging experiments were also excluded, resulting in a final list of 74 SEs (Table S3).

Demultiplexing of pooled perturbed and control cells

We measured the normalized intensity of all sample-specific oligos. Each cell was then assigned to a condition based on the fiducial Cellpose mask and the intensities of the sample-specific oligos using a nearest neighbors approach.

RNA quantification

RNA FISH data was processed and analyzed as described previously.[49,79] Images were flat-field corrected and then processed with maximum intensity projection (z axis). We used the Cellpose[73] algorithm to segment individual cells. RNA and DNA channels were aligned by maximizing the cross-correlation of the fiducial images, as described previously (see also Code Availability). RNA foci were identified with a local-maxima search function to identify the two brightest pixels for each segmented nucleus, and this region was then cropped and fit using a Gaussian PSF to identify the nascent RNA intensity (height above background from the Gaussian fit). Per gene, the distance between *cis*-linked SE alleles associated with the gene of interest and respective RNA foci were calculated, and the closest *cis*-linked SE allele was chosen as a candidate per each RNA foci for each homolog. Each homolog's candidate SE alleles were then assigned to their nearest RNA foci with the built-in MATLAB(TM) function, `matchpairs()`, and if matched, the brightness of the matched RNA foci was assigned to all associated *cis*-linked alleles for each gene. Alleles with no detectable RNA puncta within 2 μm were assigned a burst intensity value of 0. The 2 μm threshold serves to match nascent transcripts with SEs from the corresponding maternal or paternal allele and to mitigate the effects of background signal in the RNA channel. It should not be interpreted as action at a distance, as RNA nascent transcripts are flexibly tethered to the gene body, and an SE can diffuse away from the promoter in the few minutes it takes to produce a detectable nascent transcript. However, target genes cannot readily separate more than 2 μm from their associated SEs, which are typically <200 kb distal for these genes.

Sox2 SCR distance analysis

Data were downloaded from the 4DN data portal for Huang et al. 2021,[42] https://data.4dnucleome.org/publications/4f766397-5ead-416e-b891-1c95391dcd77/#expsets-table, and also collected by us as described previously.[39] The 3D positions of probes corresponding to the start, middle, and end of the *Sox2* control region super-enhancer from the unmodified allele were identified and compared.

ORCA data analyses

Single-cell contact analyses for SE clustering

For each cell, we computed the distance from all SE alleles to all SE alleles as a matrix, and thresholded this matrix at a specified physical distance (e.g., 200 nm) to determine single-cell SE contact maps. These single-cell contact maps were then used for

calculating the number of contacts a particular SE allele made with any other SE allele. The single-cell distance matrix was also used for detection efficiency calculations as pairwise distances indicate valid detection and 3D localization of SE alleles.

Frequency of SE hub sizes per SE

From the single-cell SE allele contact maps, we calculated the frequency of hub sizes of 1 (alone), 2 (pairwise), or 3+ (3+ multiway) for each SE allele, normalized by the total number of cells in which that SE allele was detected.

Average SE hub size per SE

Using the single-cell SE allele contact maps, we calculated the average SE hub size per SE allele across all cells and normalized this by the detection frequency per SE allele (overall median detection efficiency >90%, see Figure S1E).

95th-percentile hub size per SE

The 95th-percentile SE hub size was computed as for the average SE hub size per SE but reporting the 95th percentile largest cluster size across all cells per SE allele.

Simulation of uniform, random distribution of SEs

We computed *r_ave*, the average radii of the nuclei in our study from the nuclear data segmented by Cellpose (see above). We then uniformly distributed points throughout a sphere of this radius as follows: *theta* = 2**pi**rand, *phi* = acos(1–2*rand), *r* = *r_ave**rand ^($^1/_3$) where rand is a random real number in [0,1], drawn 724 times (once for each detected SE) per cell, for a total of 4000 cells. The spherical coordinate vectors were converted into Euclidean basis vectors *x,y,z*, using the standard definitions, *x* = *r**sin(*phi*)*sin(*theta*); y = *r**sin(*phi*)*cos(*theta*), and *z* = *r***cos*(*phi*). From these vectors we computed the distance maps for all 4000 cells using the MATLAB function *randMap* = squareform(pdist(*xyz*)).

Entropy

Entropy per SE allele was computed as *entropy* = -sum{ *p(e)* $\log_2$(*p(e)*)}, where the probability distribution of contacts *p(e)* across the set of SEs was computed by dividing the number of times each SE contacted each other SEs by the total number of times those SEs were observed and normalizing this distribution such that sum{*p*(*e*)} = 1. As noted in the text, if *p(e)* is a flat distribution (with equal probability of encountering any SE) this function reaches its maximum value at $\log_2$(N), where N is the total number of SE alleles. An SE that always contacted the same other SE, if it contacted any at all, the entropy function goes to 0.

Cooperativity

For all possible SE allele triplets, A, B, and C, we computed the number of cells in which A and B, and B and C were each observed within a contact threshold and normalized by the total number of cells in which all of these pairs were both detected. We call this the observed proximity frequency. We then computed the expected A–B-C frequency assuming that A-B and B-C proximity are independent of each other, by computing the product of the A-B and the B-C proximity frequencies. We then computed the observed A-B-C contact frequency by asking how many cells actually had A-B and B-C proximity, compared to the total number of cells in which A-B, and B-C were observed, respectively. We defined the cooperativity as the $\log_2$ ratio of the observed to expected.

Odds ratio

The odds ratio provides a metric of the odds that a given exposure is associated with a given condition. The exposure was defined by a minimum SE hub size (e.g., having a cluster size of two or more SEs) across multiple hub sizes and the condition was defined by detection of a transcriptional burst (RNA FISH signal >1000 au). We then define the following variables: *a*, exposed and has the condition, *b*, exposed and does not have the condition, *c*, not exposed but still has the condition, and *d*, not exposed and does not have the condition. The odds ratio is then given by the ratio OR = (*a***d*)/(*b***c*). The confidence interval was estimated by drawing a total of 10,000 bootstrap samples by random sampling with replacement using the built-in MATLAB(^{TM}) function, `randi()`, from exposure-condition pairs. The Haldane–Anscombe correction was applied to any sample where *a*, *b*, *c*, or *d* was 0.

Bootstrapped contact maps

A total of 50,000 bootstrap samples were generated by random sampling with replacement using the built-in MATLAB(^{TM}) function, `bootstrp()`, to estimate the confidence interval for the difference in contact frequencies between perturbations and controls.

ChIP-seq analysis

The fastq files underwent quality control and trimming through the utilization of Trim Galore (parameters: -q 10). Subsequently, reads were aligned to the mm10 genome via Bowtie 2[75] (parameters: -p 4 –very-sensitive). Following alignment, the reads were sorted and deduplicated using samtools. Heatmaps were generated using deeptools, utilizing normalized bigwig files as input. Peaks were called using Macs2[78] with a significance threshold set to a q-value of 0.05. Raw reads covering all super-enhancer regions were calculated using samtools for all ChIP-seq data and then normalized for subsequent analysis.

Processing Hi-C, SPRITE and GAM for ORCA validation

Pre-processed Hi-C contact-read-pairs per chromosome, were downloaded from GEO: GSE161259, and the filtered reads aligning within the 15 kb intervals of our SE regions were extracted for comparison.[41] GAM data, pre-processed per chromosome at 30 kb bin resolution, were downloaded from GEO: GSE64881.[17] The bin with the largest overlap to our SE regions was extracted and used in the comparisons. SPRITE, pre-processed per chromosome at 20 kb bin resolution, were downloaded from GEO: GSE114242.[18] The bin with the largest overlap to our SE regions was extracted and used in the comparisons. Pearson's correlation was computed using the built-in MATLAB(^{TM}) function, `corr()`, and used to calculate the correlation between contact frequencies

from ORCA at multiple contact thresholds with contact frequencies from Hi-C, GAM, and SPRITE. The built-in MATLAB(TM) function, `randperm()`, was used for random sampling of cells from ORCA data without replacement.

DamID analysis

DamID data pre-processed to 10 kb bins was downloaded from GEO: GSE181693.[72] Normalized LAD scores for untreated samples ("PT_0h") were extracted in 30 kb windows around the bin with the largest overlap to each of our SE regions. Larger windows were included to reduce the effects of data sparsity in the DamID, where resolution is limited by the sequence specificity of the Dam and by the tendency of all SEs to avoid LADs.

RNA & DNA SPRITE analysis

RNA & DNA SPRITE clusters were downloaded from GEO: GSE151515.[31] The resulting data tables were imported into MATLAB for further processing. Clusters containing any of the speckle associated RNAs, Malat1, U1.snRNA, U2.snRNA, U4.snRNA, or U6.snRNA were extracted. For each of these speckle-containing clusters we computed the total number of DNA loci in the cluster. Clusters containing over 100 DNA mapped loci were excluded in order to avoid processing of large fragments containing substantial fractions of nuclei or whole nuclei. The genomic identity of speckle-associated DNAs was mapped genome wide in 250 kb bins, summing all unique reads in each bin.

Genomic distance analysis

Weighted genomic distance

The genomic separation between a particular SE, *i*, and all other *cis* SEs, *j*, was calculated with the following formula: $D_i = \Sigma_i(1/d_{ij})$, where d_{ij} is the linear genomic distance between SEs *i* and *j*.

SE per TAD

Topologically associating domain (TAD) boundaries were obtained from a previous study using E14 mESCs.[41] The number of SEs per TAD were quantified by intersecting the 15 kb regions around each SE with TAD boundaries using bedtools and then assigned to each respective SE.

Identifying SEs in cerebellum

Super-enhancers were identified using a methodology adapted from the ROSE (Rank Ordering of Super-Enhancers) algorithm.[20] Briefly, H3K27ac ChIP-seq peaks from adult (2-month) mouse cerebellum (ENCODE experiment: ENCSR000CDC, GEO: GSM1000097) were lifted over to the mm10 assembly via CrossMap (v0.7.0). The remaining peaks were stitched into larger domains if they were within 12.5 kb of each other (GENCODE vM32 assembly and bedtools v2.31.1 were used). For each stitched enhancer region, the total integrated H3K27ac signal was quantified and each region was ranked, and a set of 362 ranked regions were chosen as cerebellum SEs (Table S2).

seqFISH+ data analyses

Processing two-layer DNA seqFISH+ probe data

Two-layer DNA seqFISH+ data was downloaded from Zenodo (https://doi.org/10.5281/zenodo.7693825).[34] For each of the 362 mouse cerebellum SEs, the closest matched 25 kb probe region from two-layer DNA seqFISH+[34] was identified based on the overlap with each 15 kb SE region. The xyz measurements for these matched regions was converted to 3D distance measurements and analyzed as described in the ORCA data analyses.

Intron seqFISH+ RNA bursting frequency analysis

Intron seqFISH+ data for E14 mESC cells were downloaded from Zenodo (https://doi.org/10.5281/zenodo.7693825).[34] The bursting frequency for each gene was calculated by dividing the number of detected RNA bursting events per gene by the total number of alleles, determined by number of cells * 2 to account for homologous alleles. The genes were then filtered to include only the SE-associated gene names from our dataset.

Statistical analysis

The significance between contact frequency distributions (Figure S6B) was evaluated using a two-sided Wilcoxon rank-sum test. Changes in hub frequency between perturbed samples and their paired controls (Figures 4C and S6D) were evaluated for significance using a two-sided Wilcoxon signed rank test. Built-in MATLAB(TM) functions `ranksum()` and `signrank()` were used to perform the statistical analyses.

Cell Genomics, Volume *6*

Supplemental information

Single-cell 3D genome imaging shows super-enhancer hubs are rare, nonspecific, and neither necessary nor sufficient for transcriptional bursting

Derek J. Le, Antonina Hafner, Achuthan Raja Venkatesh, Tee Udomlumleart, Sadhana Gaddam, Ana Novacic, Kevin C. Wang, and Alistair N. Boettiger

Figure S1, related to Figure 1

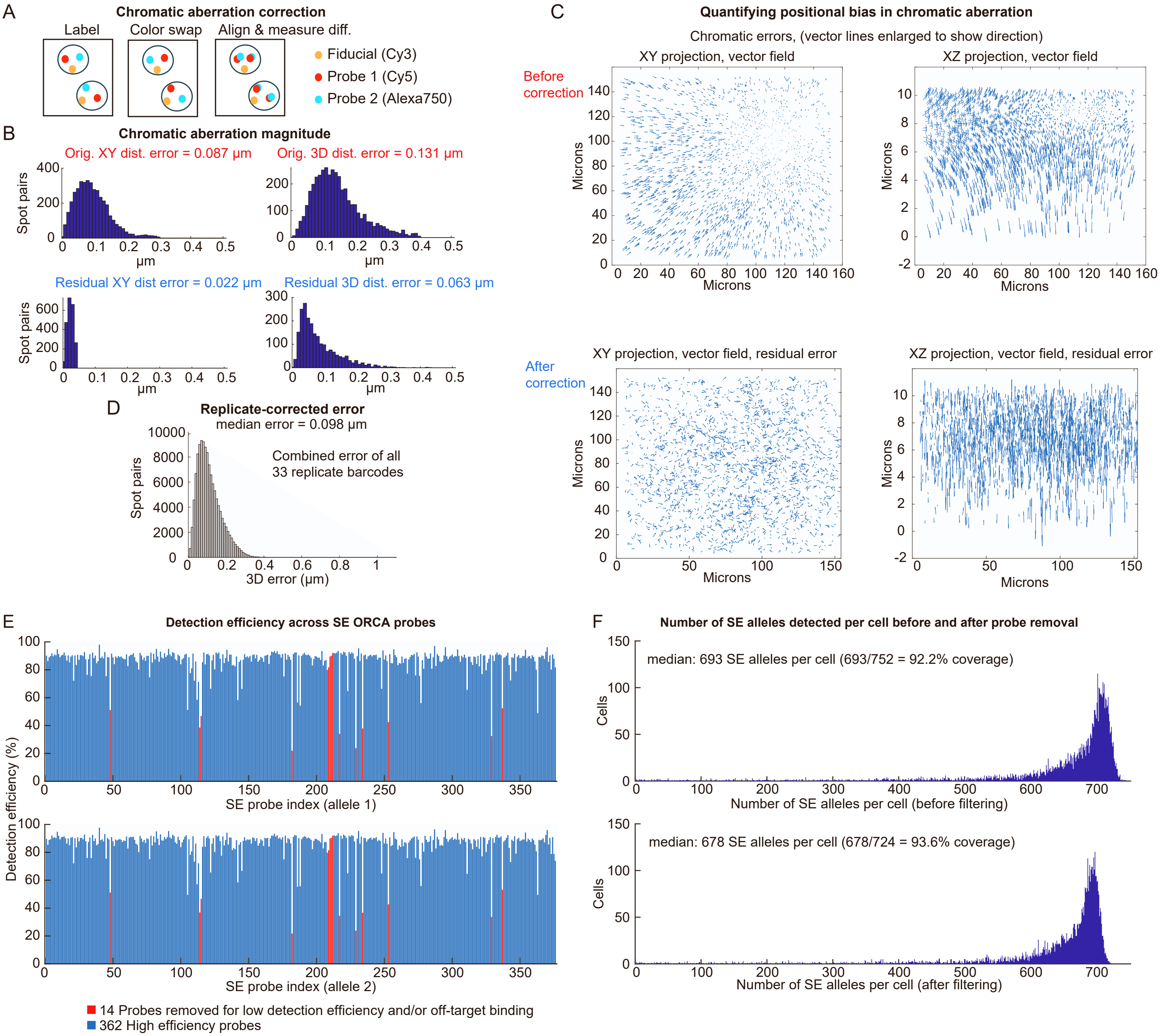

Figure S1 | Quantification of error and uncertainty, related to Figure 1. A. Schematic illustration of our approach for measuring chromatic aberration. **B.** Original chromatic aberration and corrected residual error. Note, the correction is more accurate in x,y. **C**. Field portraits of the spatial organization of chromatic aberration. Each graph represents the field of view, and each arrow starts at a position of measured chromatic aberration and points in the direction of displacement between the fluorophores. The length of the arrows has been uniformly stretched to be visible on the plot. After correction, the spatially correlated nature of the aberration has been removed. **D.** Quantification of measurement error by replicate labeling. Plot shows the aggregate of all replicate labels, performed up to hundreds of imaging rounds after the original measurement to capture the greatest extent of potential sample degradation over time. **E.** Detection efficiency per SE probe across both alleles. Probes are ordered as in Table S1. A total of 14 probes were removed for low detection efficiency and/or off target reactivity and are marked in red. **F.** Number of SE alleles detected per cell before and after removing probes in E.

Figure S2, related to Figure 1

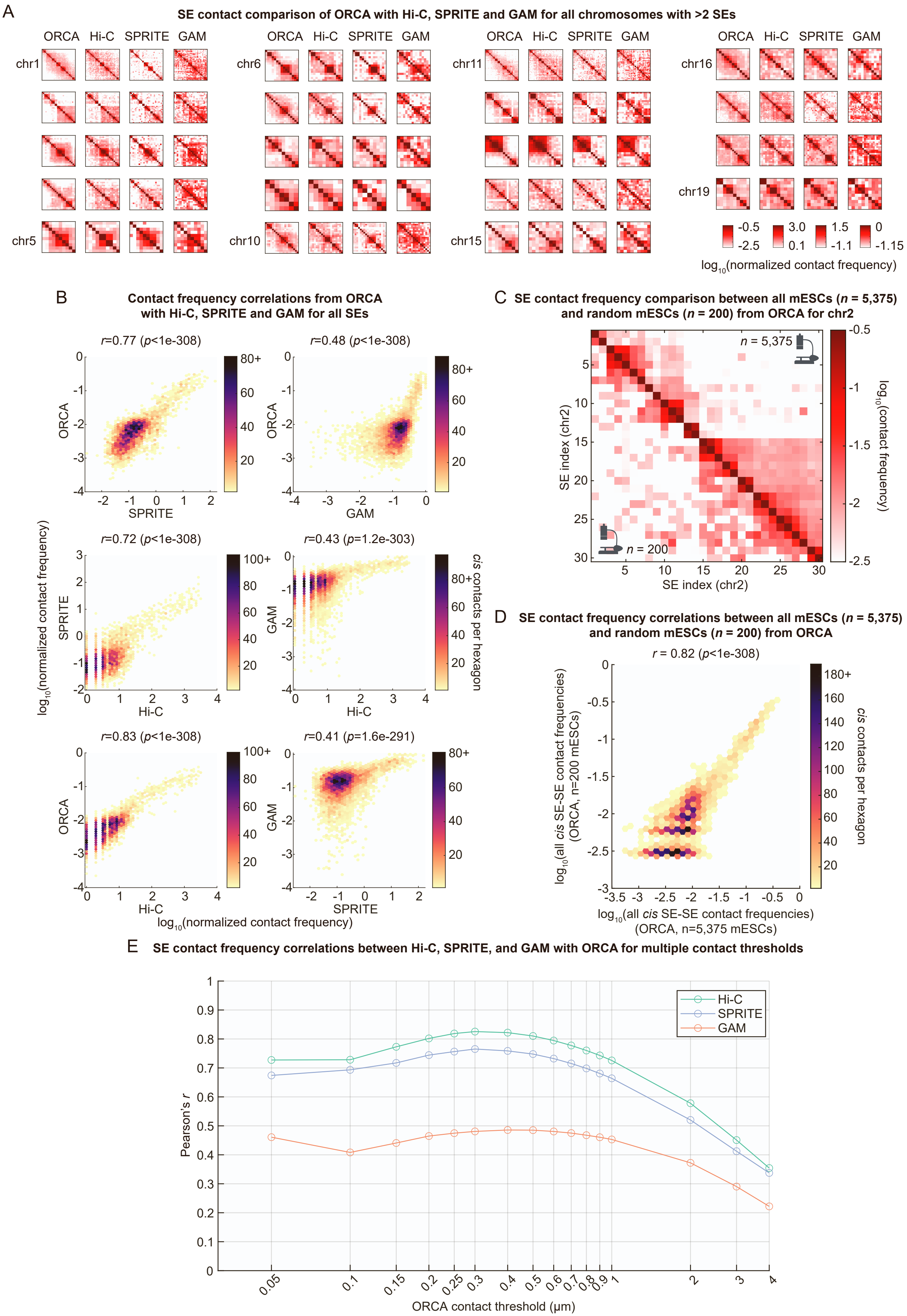

Figure S2 | Comparison of *cis* SE contacts from ORCA, Hi-C, SPRITE, and GAM, related to Figure 1. **A.** Heatmaps of the contact frequency per chromosome for ORCA, Hi-C, SPRITE, and GAM. ChrX is not shown as it contains only 2 SEs. **B.** Scatter plots comparing the normalized *cis* SE-SE contact frequencies between all 4 methods. The Pearson's correlation coefficient, *r*, and *p*-value for the log_{10}(normalized contact frequencies) are shown above each plot. **C.** Heatmap comparing the ORCA contact frequency of SEs on chromosome 2 between all mESCs (*n*=5,375 cells) and a random subset of mESCs (*n*=200 cells), using a contact threshold of 300 nm. **D.** Scatter plot comparing all *cis* SE contact frequencies measured by ORCA between all mESCs (*n*=5,375 cells) and a random subset of mESCs (*n*=200 cells), using a contact threshold of 300 nm. Pearson's *r* and *p*-value for the log_{10}(contact frequencies) are shown above the plot. **E.** Pearson's correlation of log_{10}(*cis* SE contact frequencies) between Hi-C, SPRITE, GAM and multiple contact thresholds for ORCA. The x-axis is log_2 scaled.

Figure S3, related to Figure 2

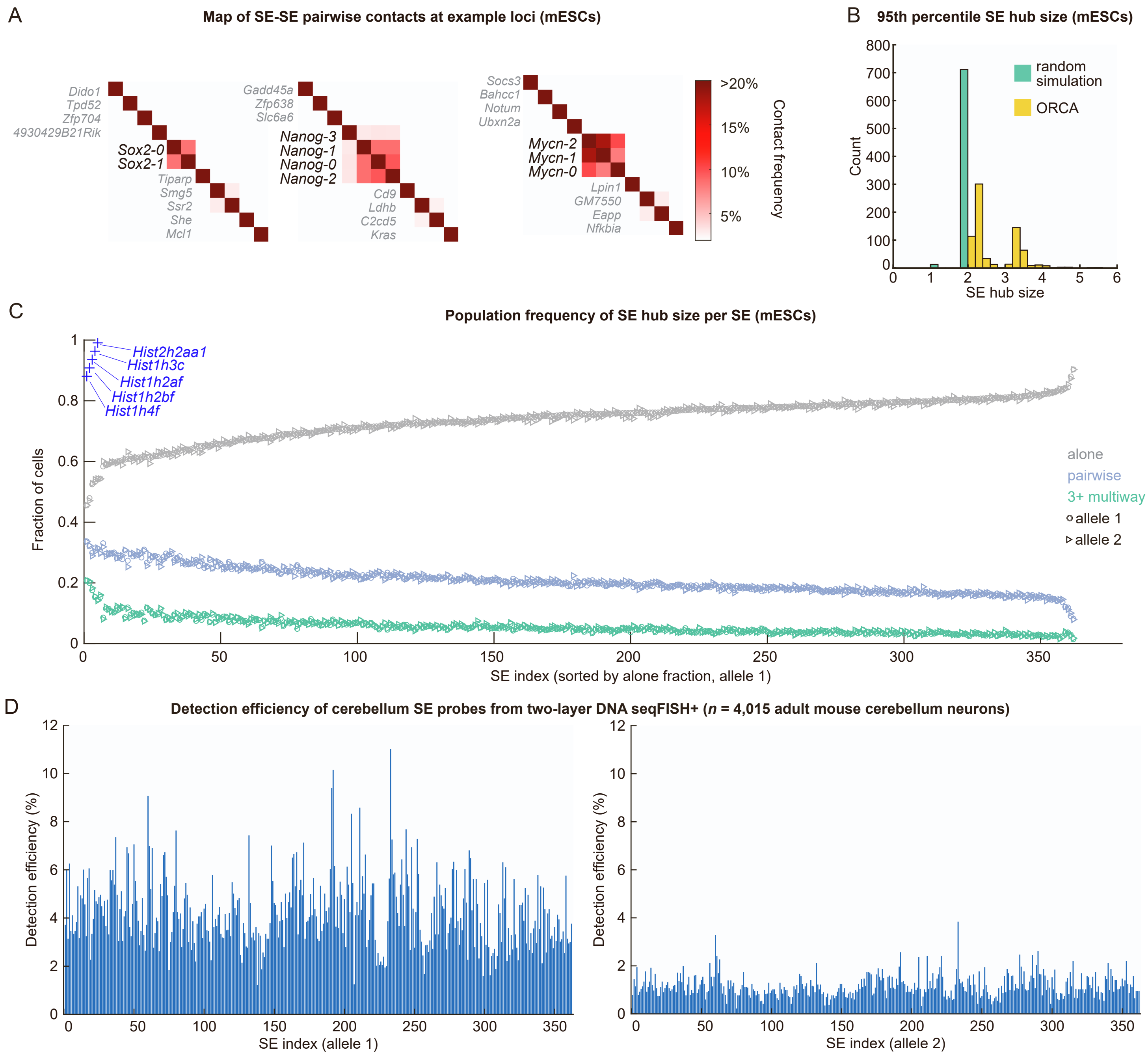

Figure S3 | Additional validation and characterization of SE interactions, related to Figure 2. A. High-frequency contacts for SEs at *Sox2*, *Nanog*, and *Mycn*. Suffixed numbers indicate SEs that were assigned to the same gene or are known to interact; a '0' indicates an SE that is at the promoter. **B.** 95th percentile SE hub size for all SEs detected by ORCA (yellow) and 724 randomly positioned SEs (green). **C**. The cell fraction for each SE when it is alone, in pairwise, or in multiway (3+) hubs. SEs are rank-sorted by the alone cell fraction size of allele 1. The five SEs with the lowest alone fraction size are indicated with a blue '+' and named. **D.** Detection efficiency of two-layer DNA seqFISH+ probes for each cerebellum SE in adult mouse cerebellum neurons, separated between both alleles.

Figure S4, related to Figure 3

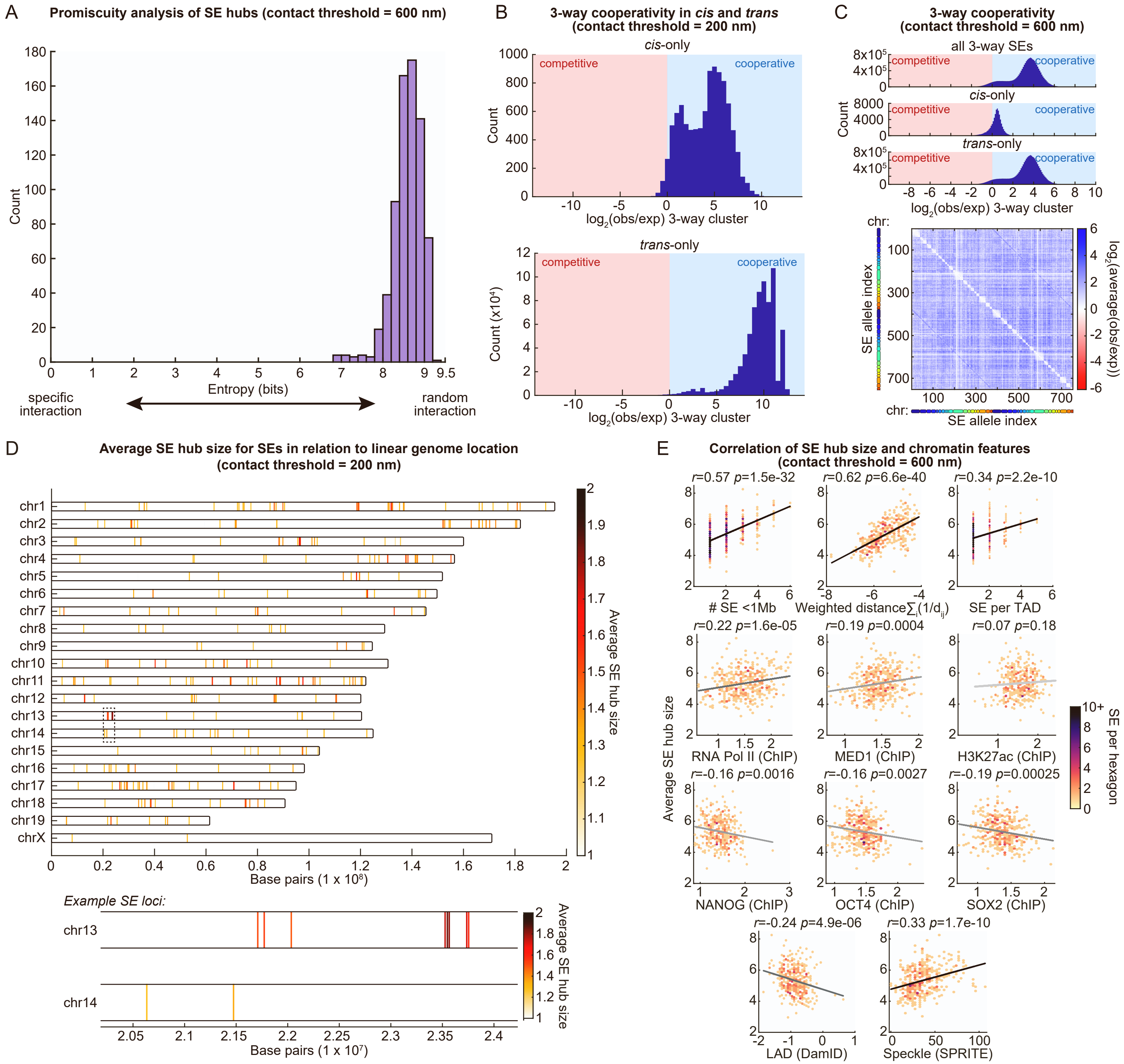

Figure S4 | Additional promiscuity, cooperativity, and correlation analyses, related to Figure 3. A. Computation of Shannon entropy for each SE over all SE hub interactions, using a contact threshold of 600 nm. **B**. Cooperativity analysis of all 3-way SE triplets ($\log_2$(O/E)), using a contact threshold of 200 nm and sorted into *cis*- and *trans*-only interactions. **C**. Cooperativity analysis of all 3-way SE triplets ($\log_2$(O/E)) using a contact threshold of 600 nm. Heatmap shows the average cooperativity score across all possible third SEs for each SE triplet combination. **D.** Chromosome maps showing the linear genomic locations for all SEs measured by ORCA and colored by average SE hub size using a contact threshold of 200 nm. Due to the large linear size of chromosomes, multiple SEs overlap each other as illustrated by the zoom-in of SEs on chr13 and chr14 (bottom). **E**. Scatter plots comparing common SE features and the average SE hub size for each SE, using a contact threshold of 600 nm. Pearson's *r* and *p*-value are above each plot. Gray/black lines are linear fits to the data, and darker line colors reflect smaller *p*-values from the Pearson correlation.

Figure S5, related to Figure 4

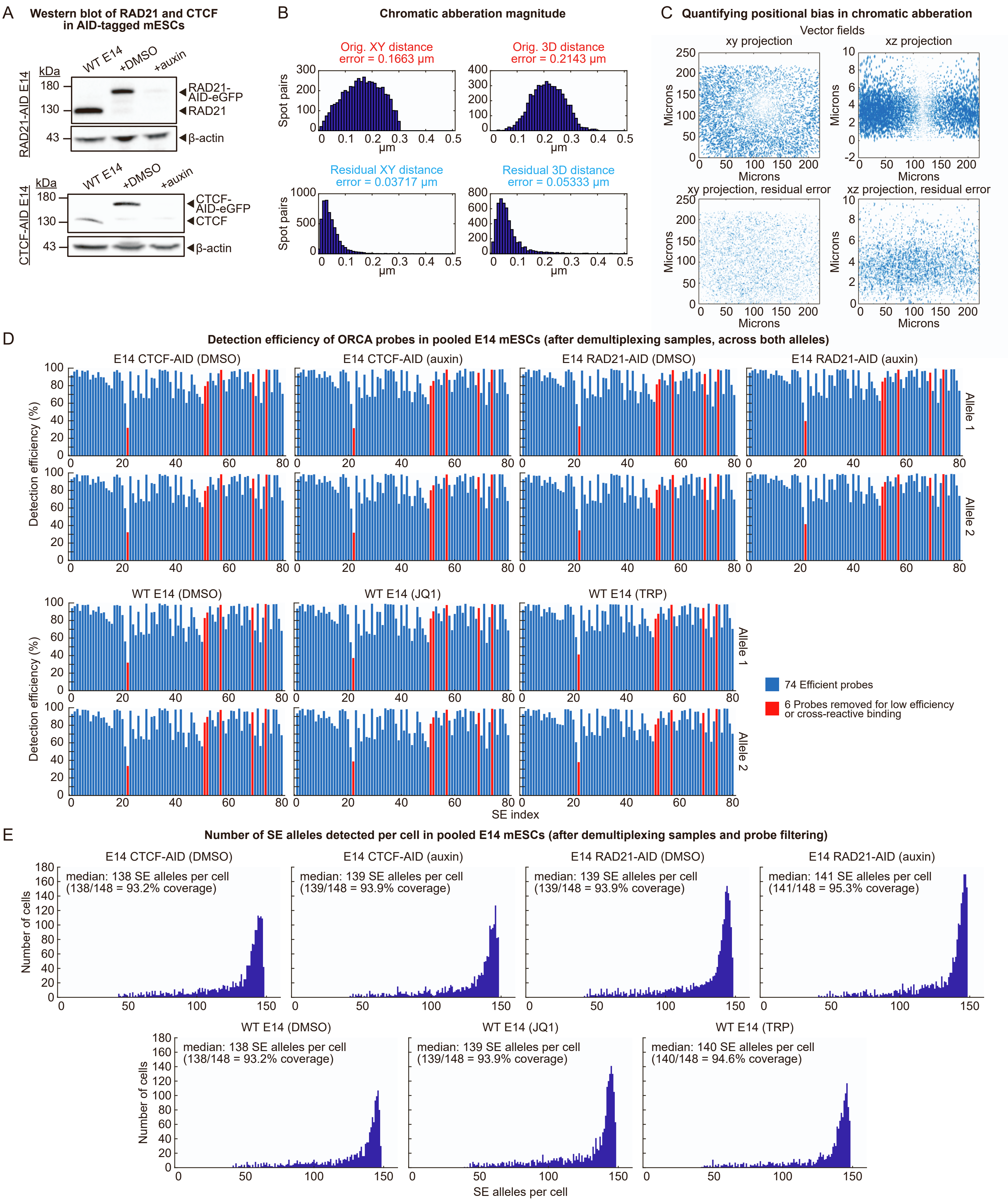

Figure S5 | Validation of perturbations on SE clustering, related to Figure 4. A. Western blot to visualize auxin-mediated degradation of RAD21 (top) and CTCF protein (bottom) in RAD21-AID-eGFP and CTCF-AID-eGFP E14 mESCs. Beta-actin was detected for loading control in both blots. **B.** Original chromatic aberration and corrected residual error. **C**. Field portraits of the spatial organization of chromatic aberration. Each arrow starts at a position of measured chromatic aberration and points in the direction of displacement between the fluorophores, and the length of the arrows has been uniformly stretched to be visible on the plot. **D**. The detection efficiency for each SE probe, for all perturbation and control treatments that were pooled for ORCA. **E.** The number of SE alleles detected per cell after removing selected probes, for all perturbation and control treatments that were pooled for ORCA.

Figure S6, related to Figure 4

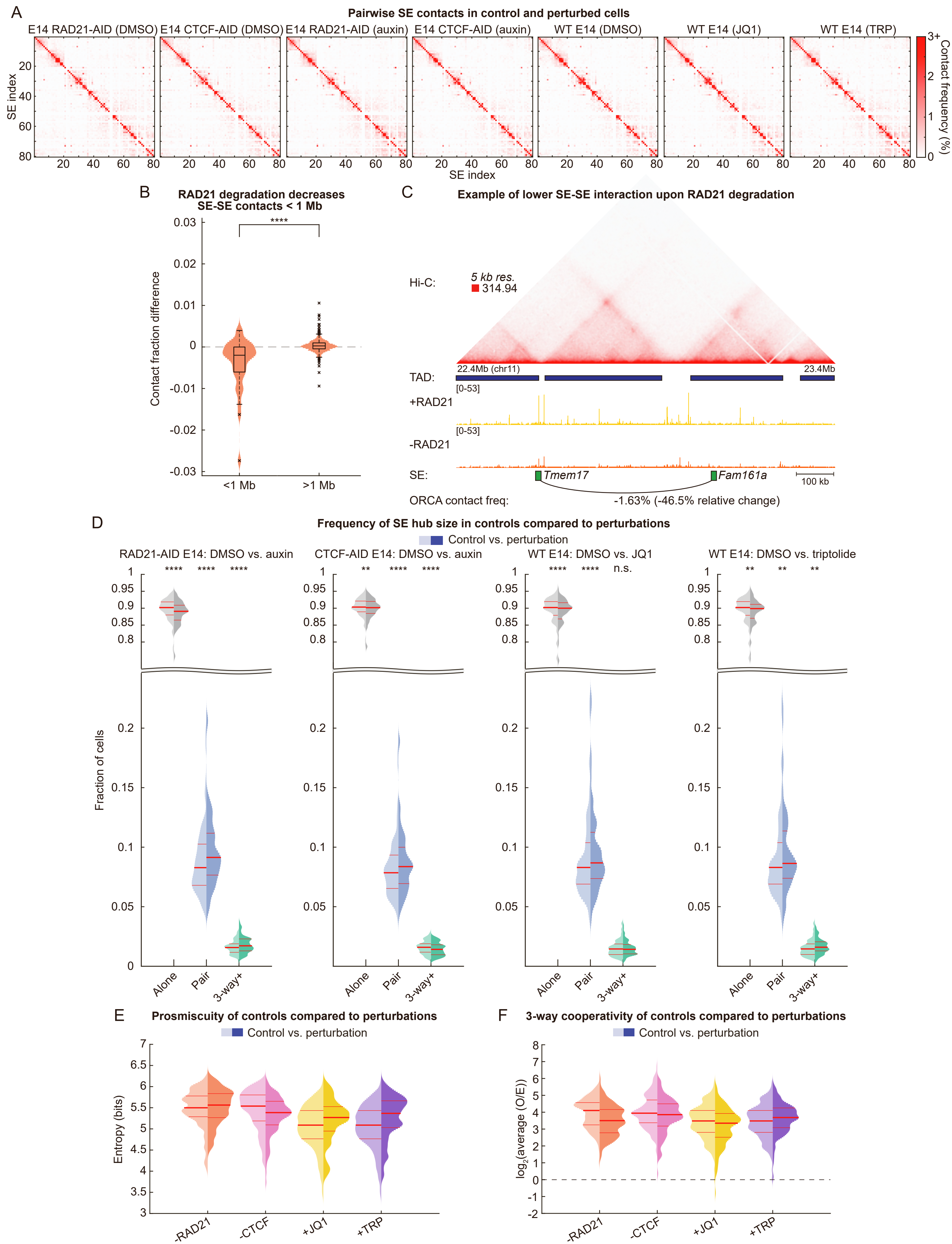

Figure S6 | Quantification of perturbations on SE clustering, related to Figure 4. **A**. Pairwise contact frequency heatmaps in perturbed and control cells for a subset of all SEs, focused on SEs from chrs 2, 3, 6, and 11 (threshold = 200 nm). **B.** Change in pairwise SE contacts binned on < 1 Mb or > 1 Mb genomic separation upon RAD21 degradation. The boxplots show the first quartile, median, and third quartile, and whiskers are 1.5 times the interquartile range. Outliers are indicated by an x. (****, $p < 0.0001$; Wilcoxon rank sum test). **C.** Change in contacts between SEs assigned to *Tmem17* and *Fam161a* in context of mESC Hi-C (Bonev et al. 2017 [S1]), mESC TADs (Bonev et al. 2017 [S1]) and mESC RAD21 ChIP upon AID-mediated degradation (Hsieh et al. 2022 [S2]). The change in the absolute and relative contact frequencies upon RAD21 degradation, measured by ORCA, is shown below the arc. **D**. The cell fraction for each SE when it is alone, in pairwise, or in multiway (3+) hubs in control and perturbed cells. (**, $p < 0.01$; ****, $p < 0.0001$; n.s., non-significant; Wilcoxon signed rank test). **E**. The entropy score for SE alleles in control and perturbed cells. **F.** The average 3-way cooperativity score for SE alleles in control and perturbed cells. **D-F.** The middle line represents the median, and the upper and lower lines represent the 75th and 25th percentiles, respectively.

Figure S7, related to Figure 5

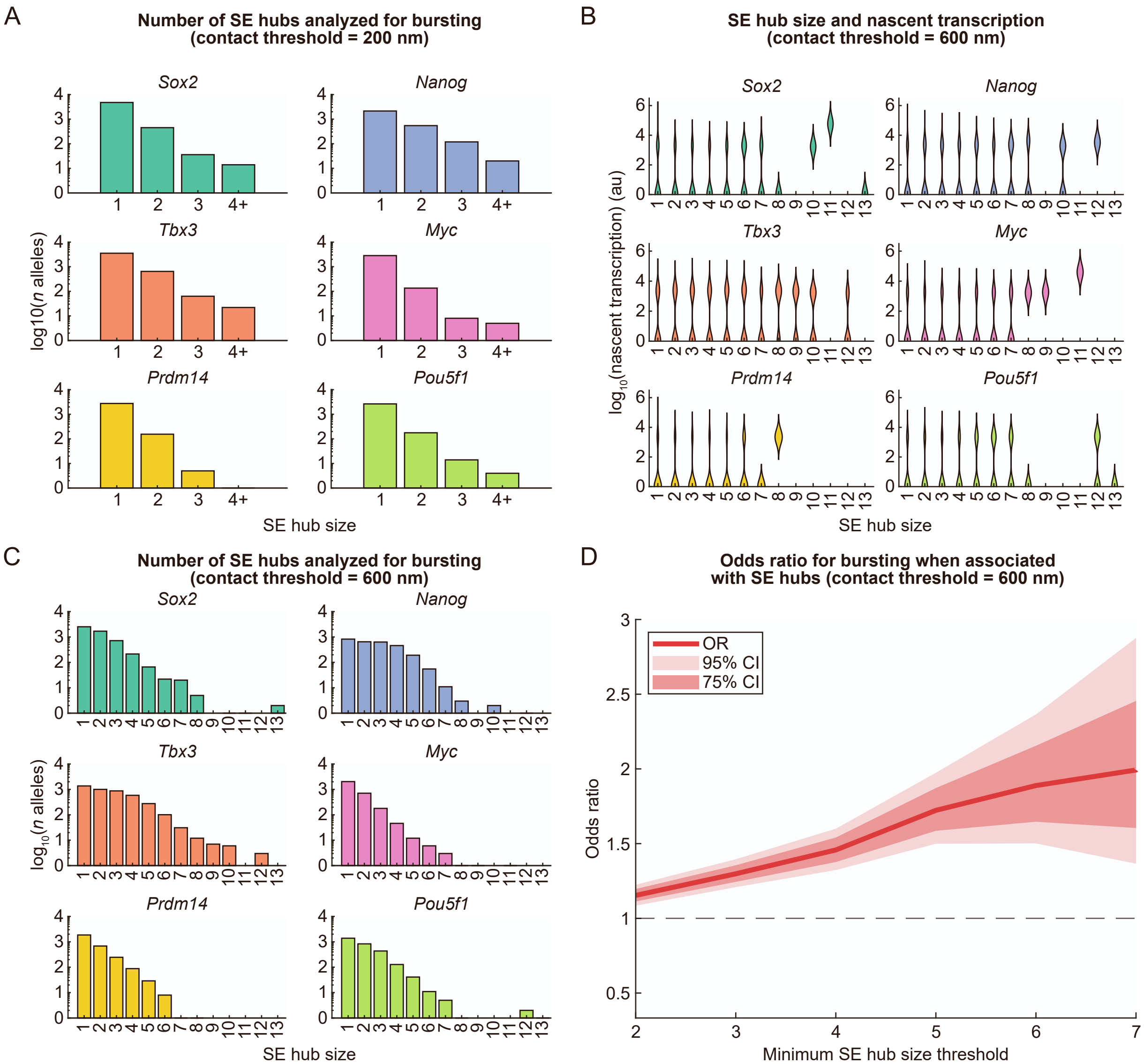

Figure S7 | Relation between transcriptional bursting and SE hubs, related to Figure 5. A. Number of alleles for each SE hub size, calculated with a 200 nm contact threshold. **B**. Distributions of nascent transcription intensity in arbitrary units (au) of the six measured genes for increasing SE hub sizes, calculated with a 600 nm contact threshold across *n*=1,727 cells. **C**. Number of alleles for each SE hub size, calculated with a 600 nm contact threshold. **D**. Odds ratio of active transcription across all of the six genes given the indicated minimum SE hub size, calculated with a 600 nm contact threshold. Shaded areas indicate the 75% and 95% confidence intervals. OR, odds ratio; CI, confidence interval.

Supplementary References

[S1] Bonev, B., Mendelson Cohen, N., Szabo, Q., Fritsch, L., Papadopoulos, G.L., Lubling, Y., Xu, X., Lv, X., Hugnot, J.-P., Tanay, A., et al. (2017). Multiscale 3D Genome Rewiring during Mouse Neural Development. Cell *171*, 557–572.e24.

[S2] Hsieh, T.-H.S., Cattoglio, C., Slobodyanyuk, E., Hansen, A.S., Darzacq, X., and Tjian, R. (2022). Enhancer-promoter interactions and transcription are largely maintained upon acute loss of CTCF, cohesin, WAPL or YY1. Nat. Genet. *54*, 1919–1932.
